# Biocompatible Guanidine-Functionalized Compounds with Biofilm and Membrane Disruptive Activity Against MRSA

**DOI:** 10.1021/acsinfecdis.5c00642

**Published:** 2025-09-15

**Authors:** Pamella Fukuda de Castilho, Luana Janaína de Campos, Audifás-Salvador Matus-Meza, Huihua Xing, Diana Liz Jimenez Rolão, Fernanda Galvão, Fabiana Gomes da Silva Dantas, Rongguo Ren, Cameron Dobrotka, Fábio Aguiar-Alves, Martin Conda-Sheridan, Kelly Mari Pires de Oliveira

**Affiliations:** † Faculty of Health Sciences, Federal University of Grande Dourados, Dourados, Mato Grosso do Sul 79804-970, Brazil; ‡ Department of Pharmaceutical Sciences, College of Pharmacy, 12284 University of Nebraska Medical Center, Omaha, Nebraska 68198, United States; § Faculty of Biological and Environmental Sciences, Federal University of Grande Dourados, Dourados, Mato Grosso do Sul 79804-970, Brazil; ∥ College of Pharmacy, University of Nebraska Medical Center, 986125 Nebraska Medical Center, Omaha, Nebraska 68198-6125, United States; ⊥ Department of Pharmaceutical Sciences, Lloyd L. Gregory School of Pharmacy, 8527 Palm Beach Atlantic University, West Palm Beach, Florida 33401, United States; # Postgraduate Program in Applied Microbiology and Parasitology and Postgraduate Program in Pathology, Fluminense Federal University, Niteroi, Rio de Janeiro 24220-900, Brazil

**Keywords:** antimicrobial, guanidine, resistant, CA-MRSA, HA-MRSA, Staphyloccocus aureus

## Abstract

Three guanidine-functionalized 3,4-dihydropyrimidin-2­(1*H*)-imine compounds (**5a**, **5b**, **5c**) were synthesized from 3,5-diaryldiene-4-piperidone and evaluated for antibacterial and antibiofilm activity against *Staphylococcus aureus*, CA-MRSA and HA-MRSA. The compounds showed bacteriostatic effects (MICs: 2.34–4.68 μg/mL). In vitro antibiofilm potential was demonstrated by significant reductions in biomass and metabolic activity, and structural analyses via SEM and fluorescence microscopy. Ex vivo antibiofilm activity was confirmed in porcine skin model. RT-qPCR revealed downregulation of biofilm associated virulence genes, indicating a multifactorial mechanism. Confocal microscopy showed increased levels of extracellular DNA and proteins, suggesting disruption of the biofilm matrix. Membrane interaction assays demonstrated time- and dose-dependent effects, suggesting a complementary mechanism of action. Compounds **5a** and **5c** exhibited synergistic and additive effects with oxacillin. The compounds were stable intracellularly, and resistance studies revealed low induction potential. Biocompatibility was confirmed by lack of mutagenicity, hemolysis, or cytotoxicity. Moreover, in vivo efficacy was demonstrated by survival of *Tenebrio molitor* larvae infected with *S. aureus* and treated. These guanidine-based compounds are promising candidates for new MRSA drug development.

## Graphical abstract

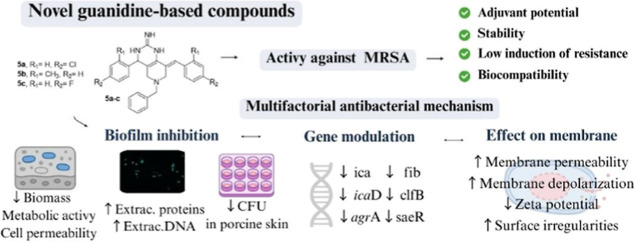

Antimicrobial resistance occurs when microorganisms develop protective mechanisms against therapeutic agents, making such agents ineffective. Antimicrobial drug resistance is one of the main global health threats of the 21st century, which is predicted to result in 10 million deaths per year around the world by 2050, with an economic impact of trillions of dollars.^1^ This problem has intensified more in the post-COVID-19 pandemic scenario because of the excessive and widespread use of antimicrobials.^2^

A particularly relevant pathogen is methicillin-resistant *Staphylococcus aureus*, MRSA, a multidrug-resistant bacteria found in both hospital and community settings. According to the Global Antimicrobial Resistance and Use Surveillance System report published by the World Health Organization (WHO) in 2022, based on data from 127 countries, MRSA was among the leading pathogens responsible for deaths associated with antimicrobial resistance worldwide.^3^ The difficulty in controlling *S. aureus* is a consequence of limited therapeutic options and the continuous emergence of resistant strains (shortly after the introduction of antibiotics in the clinic) and its remarkable ability to form biofilms, which further complicates treatment by enhancing resistance and persistence in both hospital and community environments.^4^

Furthermore, the development of new antibiotics has not kept up with the emergence of bacterial resistance. In fact, only 18 new antibiotics (from 2010 to 2021) were approved by the Food and Drug Administration. Further, most of them are the result of structural chemical modifications of currently used drugs and do not present a new mechanism of action or intracellular target.^5^ According to the latest WHO report on the clinical antibacterial pipeline, both the agents currently in development and those recently approved are insufficient to tackle the antimicrobial resistance crisis.^6^

Therefore, there is an urgent need for new therapeutic agents. The 3,5-diaryldene-4-piperidone system is considered a privileged structure due to its wide range of biological activities such as antiangiogenesis, antioxidant and anti-inflammatory properties.^7–9^ Another functional group of interest is the guanidine moiety, which is present in a variety of biologically significant molecules, notably antibacterial agents.^10,11^ In fact, others have shown that guanidine-containing molecules possess promising anti MRSA activity in vitro and in vivo.^12,13^

The protonation of the guanidine group at physiological pH, results in positively charged drugs,^11^ promoting the binding of guanidine derivatives to bacterial targets.^14^ Additionally, the positive charge can interact electrostatically with the negatively charged bacterial cell envelope, potentially leading to bacterial cell disruption.^15^

Therefore, aiming at boosting the antibacterial properties of the 3,5-diaryldene-4-piperidone compounds, we decided to incorporate a guanidine group into the design due to its antibacterial properties found in other structures.^16,17^ Specifically, we report the synthesis of three potent antibacterial 3,4-dihydropyrimidin-2­(1*H*)-imine derivatives. We evaluated their antimicrobial activity against *S. aureus* and MRSA isolates of clinical interest, and their antibacterial potential against sessile cells in an antibiofilm assay. This was further complemented by ex vivo antibiofilm evaluation in a porcine skin model. Mechanistic insights were gained from gene expression analysis via RT-qPCR, confocal microscopy and membrane interaction assays. Additionally, we report their adjuvant potential with oxacillin, stability, and ability to induce bacterial resistance. To assess biocompatibility, we conducted in vitro assays for mutagenicity, hemolysis and cytotoxicity, alongside an in vivo survival assay using *Tenebrio molitor* larvae.

## Results and Discussion

### Design and Synthesis

The synthetic strategy adopted for producing the final compounds consisted of two steps. The first included the synthesis of the 3,5-diaryldene-4-piperidone intermediates (**3a–c,** Scheme 1) following previous procedures by condensing 1-benzyl-4-piperidone (**1**, Scheme 1) with a desired aldehyde (**2a**–**c**) under basic conditions.^18,19^ The final compounds **5a**–**c** were obtained by reacting guanidine (**4**) with the intermediates **3a**–**c** under basic conditions. The compounds were purified by column chromatography and characterized by nuclear magnetic resonance and mass spectrometry.

**Scheme 1:**
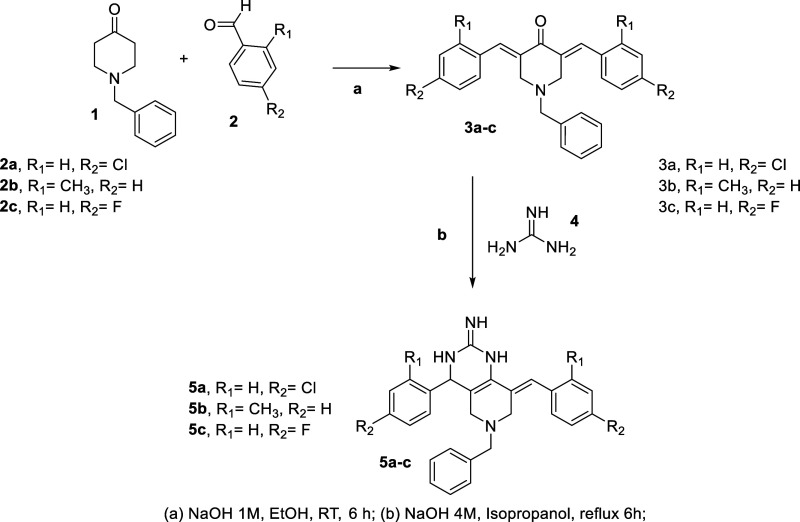
Synthetic Route to Obtain the Final Compounds

### Biological Evaluation

#### Antimicrobial Susceptibility Test

Initially, we evaluated **5a**, **5b** and **5c** against Gram-positive and Gram-negative bacteria (Table S1) and found the compounds presented activity for both types of bacteria. Our theory is the antimicrobial activity of these compounds is associated with the positive charge that the molecules possess at physiological pH.^14,15^ Positively charged molecules can readily bind to bacterial targets and/or interact electrostatically with negatively charged components of bacterial membranes such as lipopolysaccharide, lipoteichoic acid, phosphatidylinositol, and mannoproteins.^20,21^

The compounds showed higher activity against Gram-positive bacteria, particularly *S. aureus* (29213) (MIC = 2.34 μg/mL for the three molecules). Based on these findings, we tested **5a, 5b** and **5c** against strains originally isolated from the community (CA) and hospital (HA) environments. The results (Table S1) indicate that the compounds have antimicrobial potential against *S. aureus* regardless of resistance to methicillin, as the MIC values remained stable or increased by 2-fold.

#### Growth Kinetic Studies

The effect of the compounds on bacterial growth was evaluated by studying the growth kinetics of bacteria in a culture medium containing 1×, 2×, and 4× MIC of the compounds. Viable cells were counted and the results were converted to Log_10_ CFU/mL at specified intervals as shown in Figure S1. The growth kinetics of *S. aureus* (29213) and HA-MRSA exposed to the compounds differed significantly from the untreated control, which showed a progressive increase in Log_10_ CFU/mL as a function of time.

After a 2 h treatment, a decrease of Log_10_ CFU/mL was observed in both *S. aureus* (29213) and HA-MRSA strains, particularly in those treated with 4× MIC. However, it is worth noting that none of the treatments completely eliminated the cells, which highlights the bacteriostatic action of the compounds. Bacteriostatic agents inhibit growth, while bactericidal agents cause bacterial death. Both are clinically relevant, with distinct therapeutic implications depending on infection type, host immune status, and infection site.^22^

#### Antibiofilm Activity and Characterization

Biofilms are communities of bacteria living within a self-produced matrix of extracellular polymeric substances. This matrix provides, among other properties, tolerance and resistance to antimicrobials.^23^ Our study assessed the potential of the compounds against biofilms of *S. aureus* (29213), CA-MRSA and HA-MRSA. We considered the antimicrobial susceptibility results to determine 1×, 2×, and 4× MIC concentrations, which were used in the test. Following treatment, the biofilms were characterized.

Biofilm biomass was assessed using the crystal violet assay, which stains negatively charged surface molecules, viable and nonviable cells, and the extracellular polysaccharide matrix, allowing quantification of the entire biofilm.^24^ The biofilm formation and destruction assays treated with our compounds demonstrated a significant reduction in biomass on the three strains and 4× MIC treatment showed the best results (Figure S2). In the biofilm inhibition assay, molecules **5a** and **5c** demonstrated complete inhibition of biofilm biomass. In the biofilm destruction assay, the most effective molecule was **5a**, which exhibited destruction of 45.8%, 42.0% and 32.5% of the biomass of *S. aureus* (29213), CA-MRSA and HA-MRSA biofilms, respectively.

The metabolic activity of the biofilm should also be evaluated, as crystal violet is only measures total biofilm biomass, not functionality/viability biofilm.^25^ After treatment with the molecules, the metabolic activity of the *S. aureus* (29213), CA-MRSA and HA-MRSA biofilms were measured with XTT, a tetrazolium salt that is reduced by mitochondrial enzymes and presents an orange color.^26^ Our results demonstrate that all evaluated concentrations of our compounds reduced the metabolic activity of biofilms (Figure 1).

**Figure 1:**
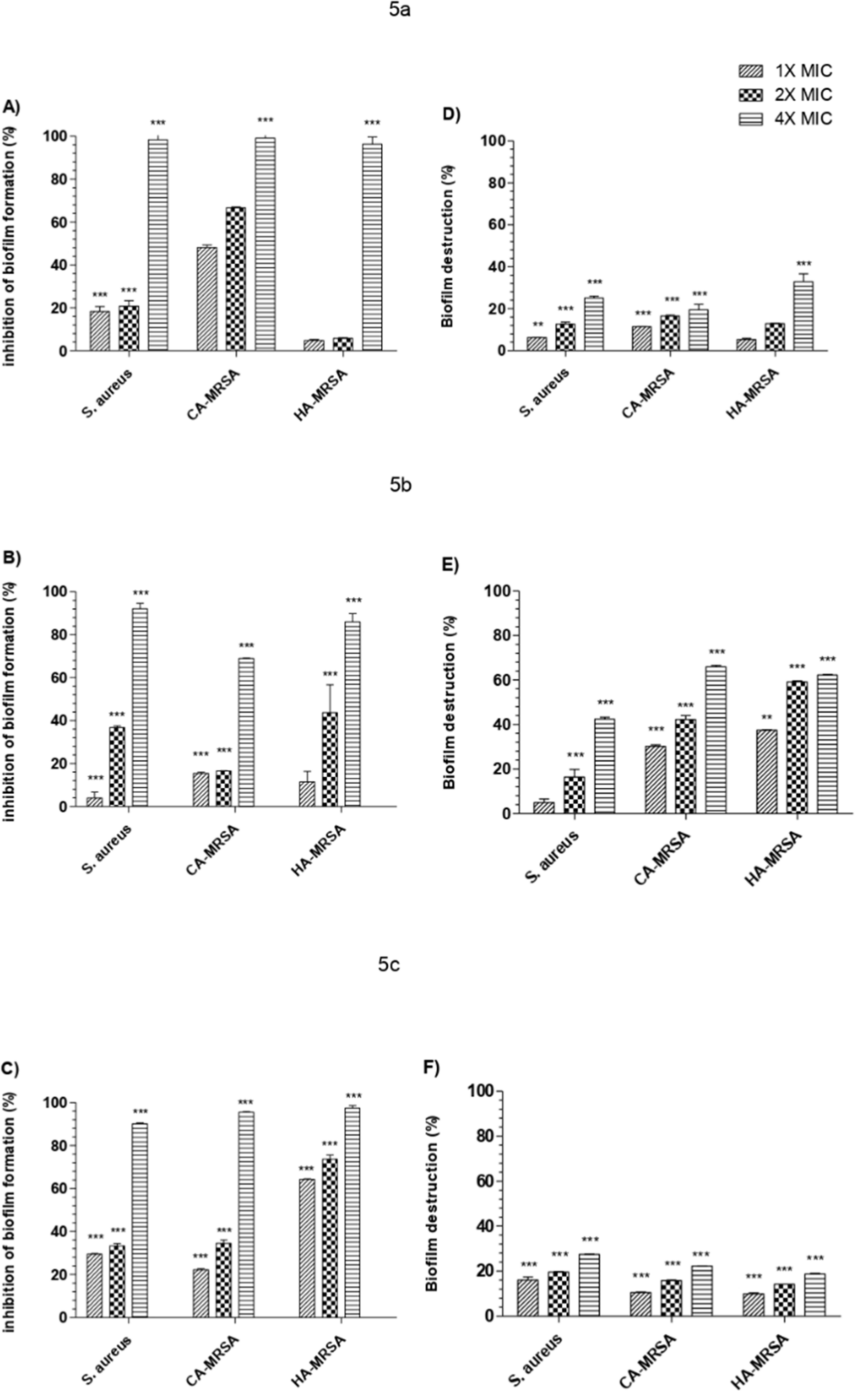
Inhibition (A–C) and destruction (D–F) of *S. aureus* (29213), CA-MRSA, and HA-MRSA biofilms assessed by XTT metabolic activity assay. **P ≤ 0.01; ***P ≤ 0.001 vs control (ANOVA, Dunnett’s test). Error bars omitted when SD < symbol size; each experiment had technical replicates.

The compounds tested in the biofilm inhibition assay reduced the metabolic activity of both CA-MRSA (99.1–15.4%) and HA-MRSA (97.5–4.8%). Notably, compounds **5c** (97.6–64.2%) and **5b** (86.1–11.5%) were more effective against hospital-associated isolates compared to community isolates. In the biofilm destruction determination, the best results were observed at the concentration of 4× MIC, with a reduction in metabolic activity of 62.3–18.7% for HA-MRSA and 65.3–19.5% for CA-MRSA biofilms. Mature pathogenic biofilms require 100–1000× higher antibiotic concentrations than planktonic cells.^27^ In our study, the compounds significantly reduced (*P* ≤ 0.001) biofilm metabolic activity at just 4× MIC in the destruction assay.

The SEM micrographs (Figure 2) indicate the compounds inhibit the formation of the HA-MRSA biofilm in comparison to the positive control (A0, not treated). The dose–response relationship was positive, increasing the evaluated concentrations resulted in a more prominent reduction of cells. At the concentration of 4× MIC of compounds **5a** and **5c** we observed 100% inhibition (Figure 2B3,D3).

**Figure 2:**
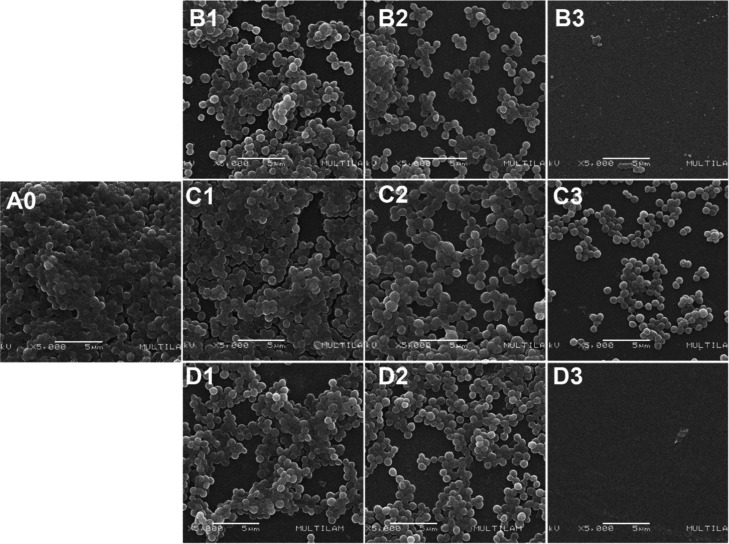
SEM micrographs of HA-MRSA biofilms from the inhibition assay, cultivated with different compound concentrations. (A0) Untreated Biofilm; (B) B1–D1 1× MIC; (C) B2–D2 2× MIC; (D) B3–D3 4× MIC. (B) Corresponds to **5a**; (C) corresponds to **5b**; (D) corresponds to **5c**.

The SEM images also allowed visualization of the effect of compounds on HA-MRSA cell integrity (Figure 3). Untreated cells (Figure 3A) showed normal morphology: smooth surfaces and well-defined round shape. Cells treated with 2× MIC (Figure 3B–D) exhibited collapse, morphological alterations, and structural damage, suggesting membrane permeabilization.

**Figure 3:**
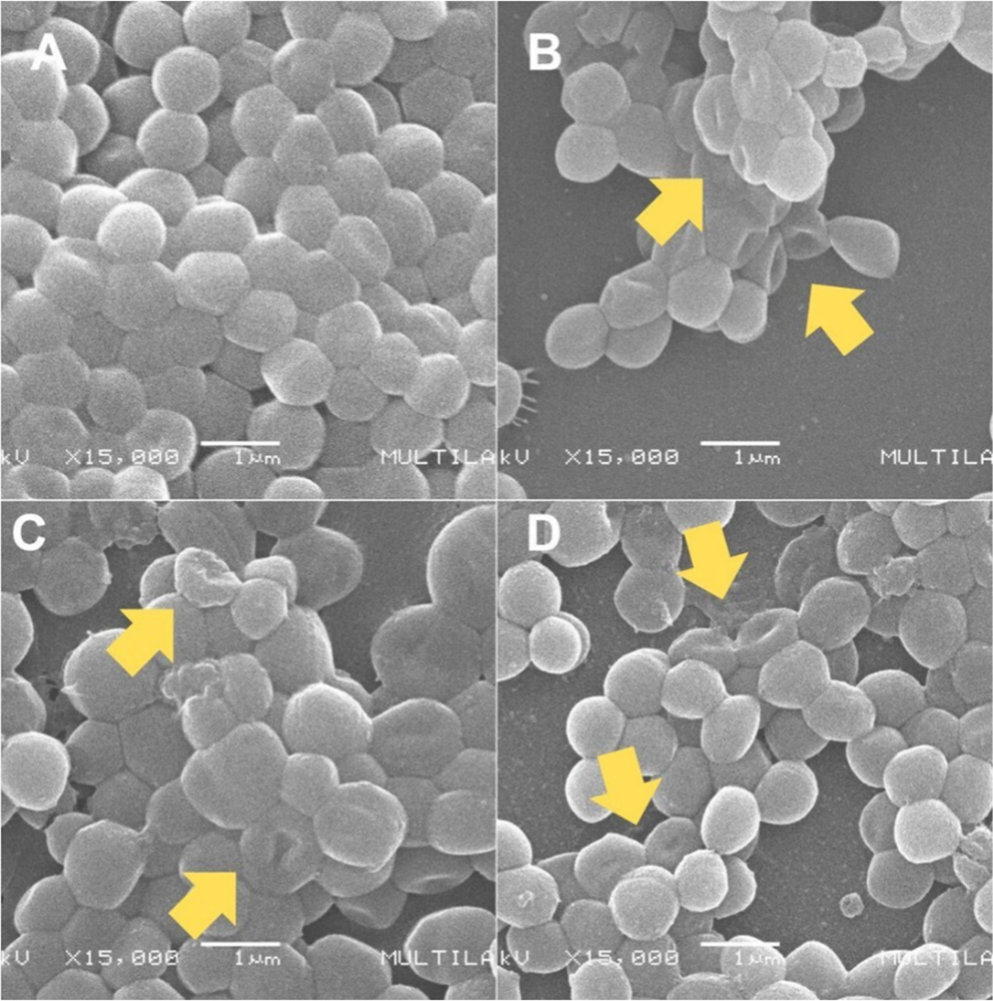
SEM micrographs showing cell integrity of HA-MRSA biofilms from the inhibition assay. (A) Untreated Biofilm; (B) 2× MIC of **5a**; (C) 2× MIC of **5b**; (D) 2× MIC of **5c**. Arrows highlight cells with collapse, morphological changes, or structural damage.

To evaluate the activity of the compounds on the bacterial cell viability of the HA-MRSA biofilm, a live/dead staining was performed. According to Figure S3, cells treated with the compounds **5a**–**c** (panels B, C and D) stained predominantly with fluorescent red, indicating membrane damage. The opposite was observed in the untreated control images, (Figure S3A), where a predominance of green fluorescence is evident, demonstrating that these cells had intact membranes. The characteristics of the dyes, which differ in spectral ranges and cell penetration capacity, suggest that **5a**, **5b** and **5c** can affect cell permeability due to the predominance of cells-stained in fluorescent red after treatment.

#### Antibiofilm Efficacy in an *Ex Vivo* Porcine Skin Model

In order to evaluate the antibiofilm activity in a more complex biological microenvironment, an ex vivo assay was conducted using a porcine skin model. The results (Figure 4) demonstrated that compound **5c** significantly inhibited biofilm formation. At a concentration of 2× MIC, the inhibition rates were 49.5%, 49.0%, and 59.0%, whereas at 4× MIC, the inhibition rates were 76.6%, 58.7%, and 76.7% for *S. aureus* (29213), CA-MRSA, and HA-MRSA, respectively. These findings corroborate the in vitro results and demonstrate that compound **5c** retains its potential to inhibit biofilm formation even in conditions that mimic a three-dimensional structure, reproducing key structural aspects of the skin.

**Figure 4:**
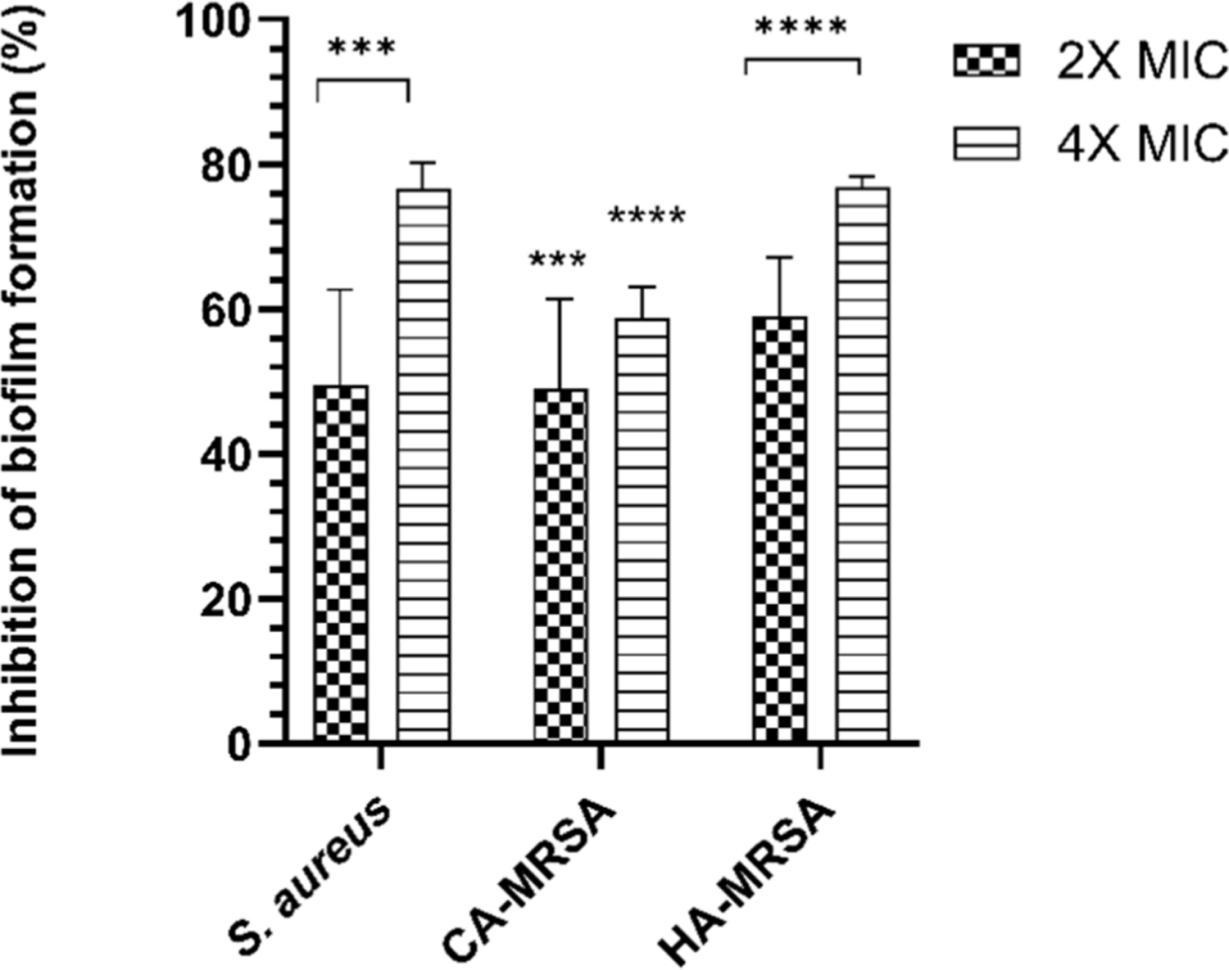
Effect of compound **5c** on biofilm inhibition of *S. aureus* (29213), CA-MRSA, and HA-MRSA in an ex vivo porcine skin model, at 2× and 4× MIC. ***P ≤ 0.001; ****P ≤ 0.0001 vs positive control (ANOVA, Dunnett’s test). Each experiment included technical replicates. Results are expressed as percentage inhibition relative to the positive control (0% inhibition).

Moreover, the porcine skin model reinforces the relevance of these findings as it is widely used due to its similarity to human skin, while also permitting the evaluation of antimicrobial activity in a biologically relevant environment that preserves, tissue complexity and reducing ethical and regulatory concerns associated with the utilization of live animal models.^28,29^ The combination of in vitro and ex vivo data strengthens the robustness of the antibiofilm activity of compound **5c** and highlights its potential for developing novel therapeutic strategies against biofilm-associated infections, particularly those caused by resistant strains such as MRSA.

#### Modulation of Gene Expression following Drug Exposure

Gene expression analysis via RT-qPCR demonstrated that treatment with compound **5a**, at different concentrations, resulted in a significant reduction in the expression of genes related to virulence, biofilm formation, and maintenance in *S. aureus* (JE2) during the treatment to prevent biofilm formation. Figure 5A–F shows a significant reduction in the relative expression levels of *ica*, *icaD*, *agrA*, *fib*, *clfB*, and *saeR* were observed compared to the untreated group. This reduction was dose-dependent, being more pronounced at higher concentrations of **5a**. Furthermore, Figure 5G compares the negative control with the combination of molecule **5a** and vancomycin, demonstrating that gene expression was also reduced following treatment, except for *agrA*.

**Figure 5:**
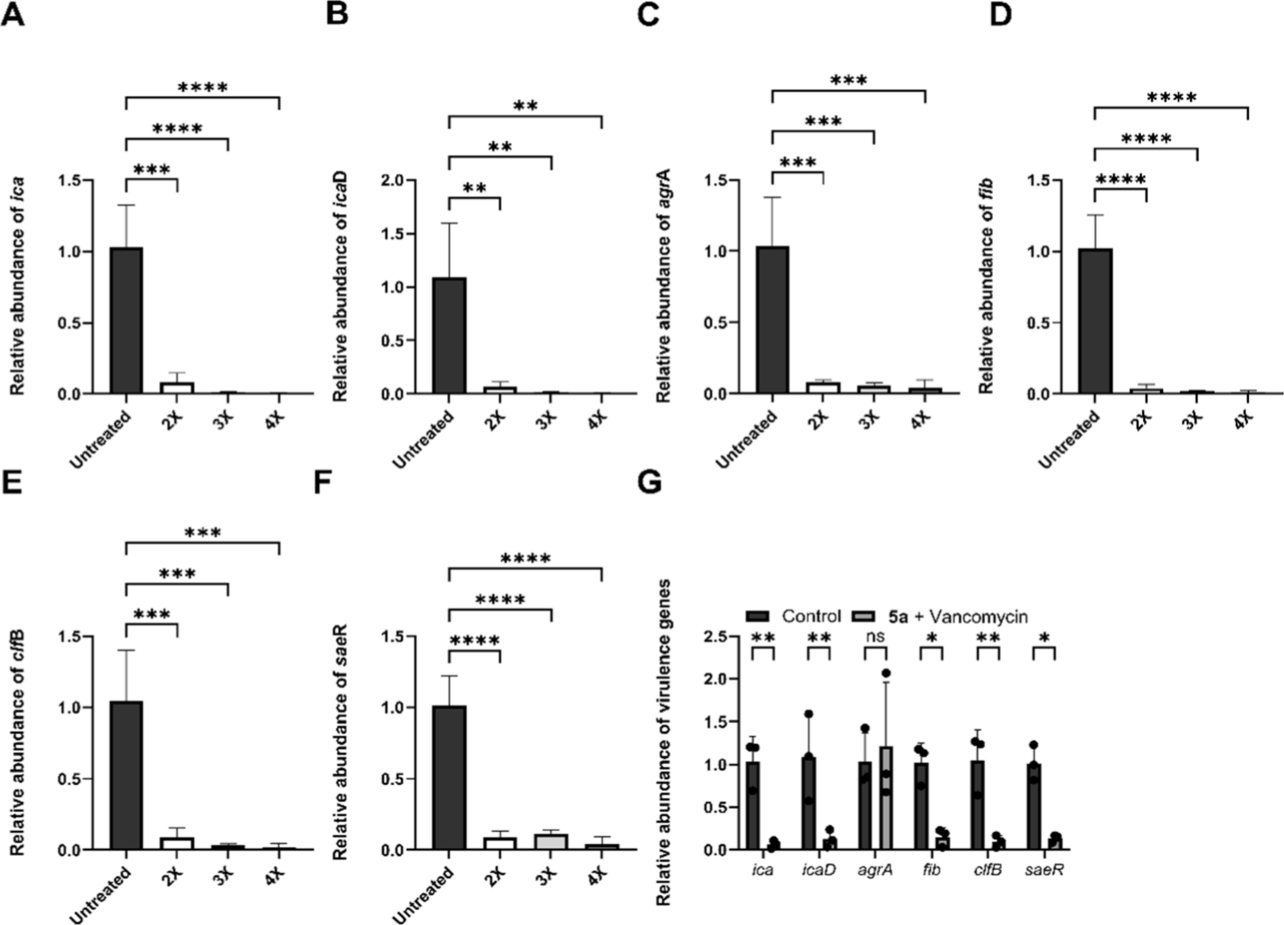
Gene expression analysis of key virulence factors in *S. aureus* (JE2) biofilms after 24 h treatment with **5a**, assessing relative changes in *ica*, *icaD*, *agrA*, *fib*, *clfB*, and *saeR* (A–F). Combination of **5a** with vancomycin (1 μg/mL) tested for all factors (G). Statistics: one-way ANOVA (Dunnett) and two-way ANOVA (Šídák), *P ≤ 0.05; **P ≤ 0.01; ns = not significant.

The *fib* and *clfB* genes encode surface proteins that play a crucial role in the initial colonization of *S. aureus*, facilitating host cell adhesion and cell aggregation. The *ica* and *ica*D genes are part of the *ica*ADBC operon, which regulates the synthesis of polysaccharide intercellular adhesin (PIA), one of the components associated with the biofilm matrix. The expression of these genes may vary among strains and environmental conditions, potentially contributing to biofilm architecture and stability. *agrA* is an essential regulator of the *agr* quorum sensing system, which governs bacterial cell communication and plays a critical role in virulence regulation and biofilm dispersion.^4,30^

The downregulation of these genes following treatment suggests that the mechanism of action of **5a** involves inhibition of biofilm formation through interference with multiple regulatory pathways, including PIA synthesis, quorum sensing, and bacterial adhesion. While this highlights a key mechanism related to biofilm inhibition, other complementary effects likely contribute to the compound’s overall antibacterial activity, as discussed in subsequent sections.

Furthermore, the reduced expression of *sae*R, a global virulence regulator, suggests that treatment with **5a** may impact the regulation of essential pathogenicity factors. The SaeRS system controls genes involved not only in cellular adhesion but also in immune evasion and toxin secretion, including cytotoxins.^31^ Therefore, *sae*R suppression compromises the ability of *S. aureus* to establish persistent infections by affecting both host cell adhesion and the expression of proteins essential for bacterial survival and virulence.

Vancomycin is an antibiotic with limited efficacy against biofilms.^32^ However, its combination with **5a** indicate a significant reduction in the expression of *ica*, *ica*D, *fib*, *clf*B, and *sae*R, suggesting that **5a** may enhance vancomycin activity against biofilms. Conversely, the absence of a significant reduction in *agr*A expression under this condition suggests that vancomycin may modulate *S. aureus* (JE2) response to **5a** by specifically interfering with this regulatory pathway.

#### The Biofilm Composition is Altered with **5a** Treatment

After observing a difference in the expression of biofilm-associated factors and significant changes in biofilm biomass when treated with **5a**, we hypothesized that the structural components of the biofilm would likely be altered. To test this, we performed laser scanning confocal microscopy, specifically targeting the biofilm matrix protein and extracellular DNA (eDNA). Specifically, we used SYPRO Ruby Red protein stain and membrane-impermeable TOTO-1 iodide to stain matrix proteins and eDNA, respectively.

We found that when *S. aureus* (JE2) biofilms were treated for 24 h at a concentration three times higher than the MIC of **5a**, there were changes in the amount of eDNA and protein present compared to the control (Figure 6A–F). DMSO was used as a control for viability and staining, which provided minimal intracellular signal for DNA or protein, while the biofilm matrix showed a robust signal (Figure 8A–C). Interestingly, **5a** shows a strong fluorescence signal for both DNA and protein inside all *S. aureus* (JE2) cells present, as well as a significant amount of eDNA and protein in the matrix (Figure 6D–F) likely attributed to cell death and leakage of intracellular DNA.

**Figure 6:**
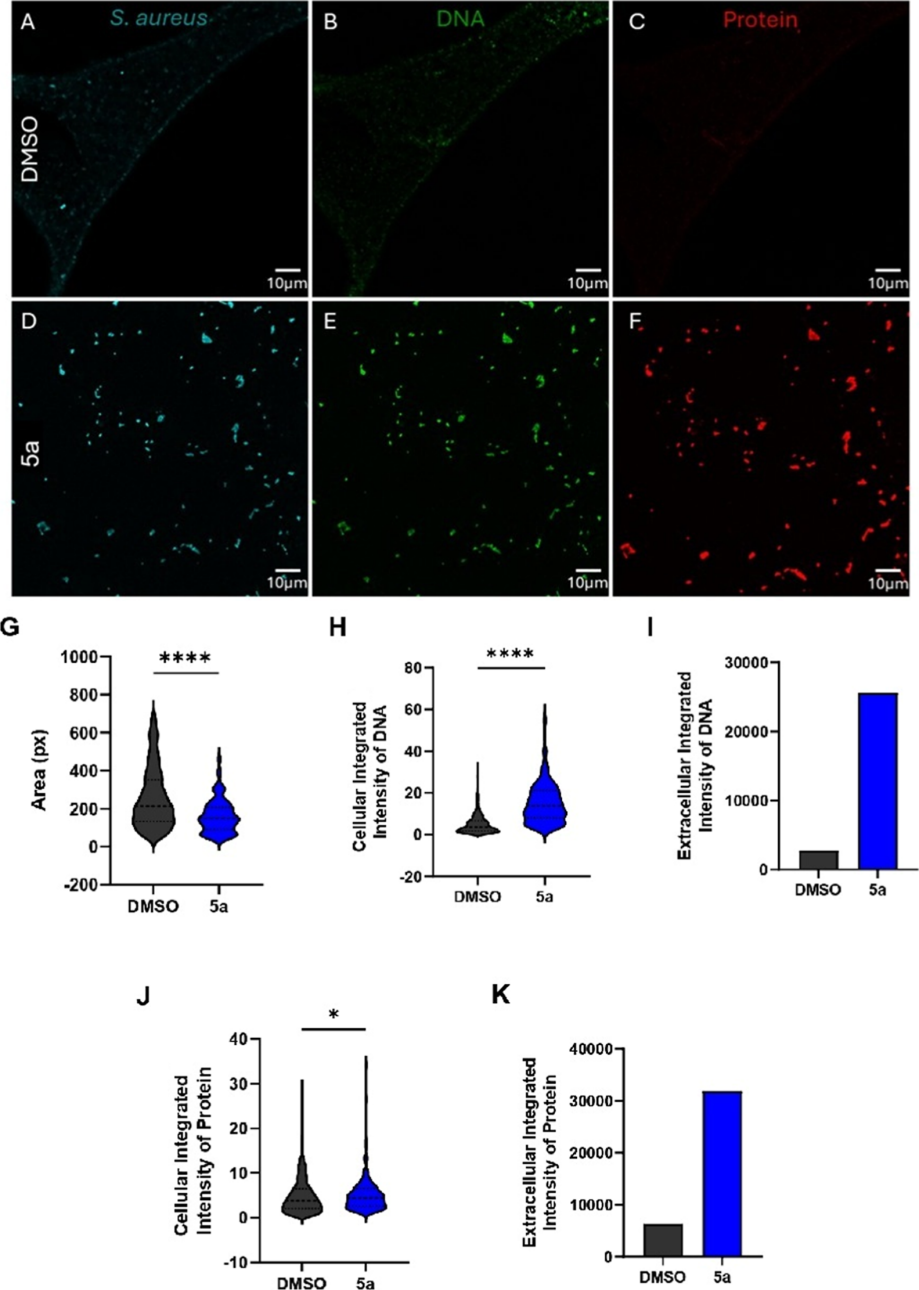
DMSO control (A–C) and **5a** (D–F) at 3× MIC with equivalent DMSO against *S. aureus* (JE2) biofilms. Wheat Germ Agglutinin Alexa Fluor 647 labeled cells (A,D); Toto-1 labeled extracellular DNA (B, E), including eDNA in the biofilm matrix; SYPRO Ruby Red labeled matrix proteins (C,F). Area and integrated intensity of DNA/protein were quantified with CellProfiler (G,H,J), and extracellular intensity measured in the same pipeline (I,K).

To further demonstrate the efficacy of the compound, cell area, integrated density of TOTO-1 (DNA), extracellular integrated density of TOTO-1 (DNA), intracellular integrated density of SYPRO (protein), and extracellular integrated density of SYPRO (protein) were quantified using mean intensity projections and a customized CellProfiler pipeline. After quantification, we found that the cell area treated with **5a** was significantly smaller than the DMSO control (Figure 6G). The integrated density of DNA stained with TOTO-1 was significantly higher when cells were treated with **5a** compared to the DMSO control (Figure 6H). Moreover, **5a** appears to increase the amount of eDNA present in the biofilm matrix, likely coinciding with its membrane-disrupting mechanism of action (Figure 6I). When observing the amount of protein stained inside the cells, the integrated intensity of SYPRO-stained intracellular protein was significantly higher in the **5a**-treated group compared to the DMSO control (Figure 6J). Similarly to extracellular DNA, extracellular protein levels were also elevated when *S. aureus* (JE2) cells were treated with **5a** (Figure 6K).

#### Effect on the Bacterial Membrane

Considering previous studies that have demonstrated the activity of guanidine-containing compounds on bacterial membranes, we also investigated the potential of **5a**, **5b**, and **5c** in this context.^15,18,19^ First, the membrane permeabilization was evaluated using the DNA-binding dye PI (Figure 7A). This dye, due to its strong binding to DNA, shows an increase in fluorescence when it enters the compromised bacterial cell as it readily interacts with intracellular DNA.^33^ As it can be observed in Figure 7A, in general, the compounds did not induced relevant permeability changes up to 4× MIC (MIC = 2 μg/mL) in *S. aureus* after 30 min of treatment, as evidenced by no relevant change in the PI fluorescence (Figure S4 displays statistical analyses of all tested concentrations over time).

**Figure 7:**
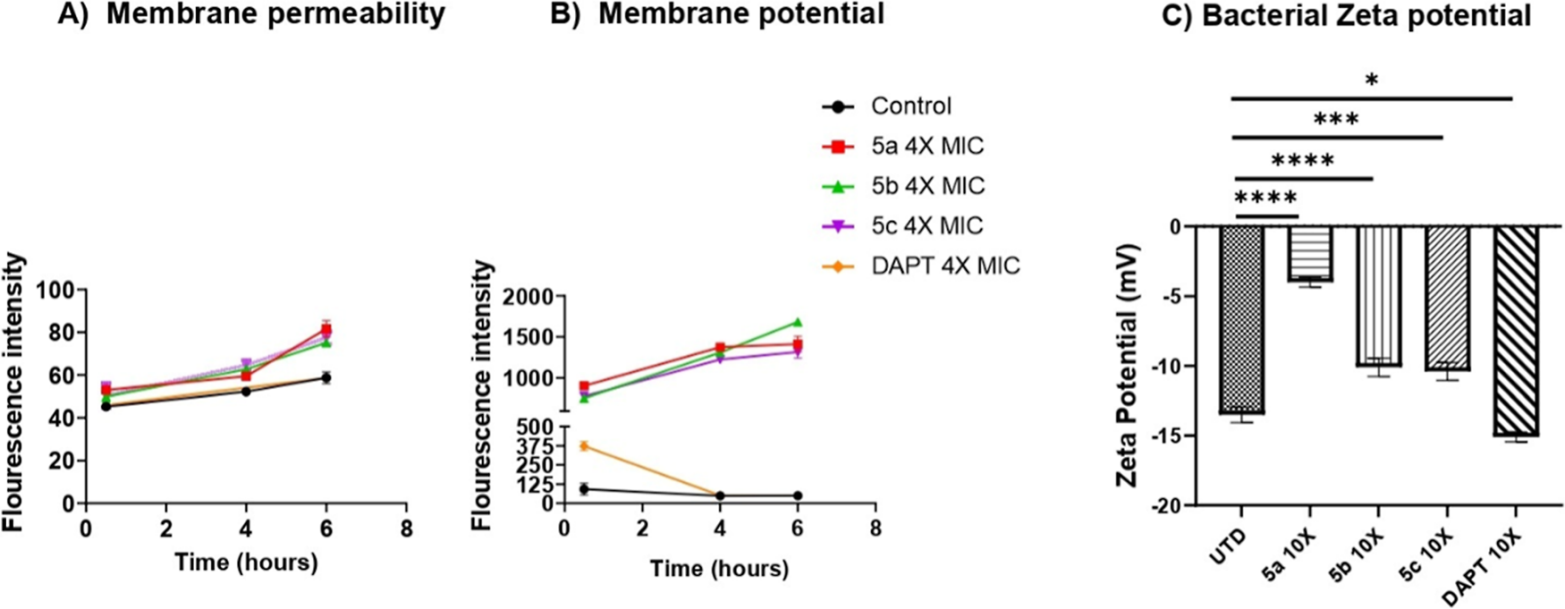
Membrane activity of the studied compounds in *S. aureus* JE2. (A) Inner membrane permeabilization via PI uptake; (B) membrane potential by voltage-sensitive dye [DiSC_3_(5)]; (C) zeta potential changes after guanidine treatment. Error bars = SEM; *n* = 3 biological replicates. *P ≤ 0.05, ***P ≤ 0.001, ****P ≤ 0.0001; (one-way ANOVA, Dunnett’s test).

Since changes in membrane permeability are time-dependent, we treated the bacteria for a longer period.^34^ As shown in Figure 7A, after 4 h and especially at 6 h of drug treatment, an alteration in the PI fluorescence was seen for the 4× MIC treatment. Thus, the data indicates the compounds can disturb the permeability of the membrane in a time and concentration dependent manner (Figure S5 shows data for other concentrations).

It is accepted that changes in the membrane permeability induced by drugs are capable of inhibiting and killing bacteria by dissipating the transmembrane potential.^35^ Therefore, we investigated the cytoplasmic membrane depolarization effect of our compounds using the voltage-sensitive stain [DiSC3(5)]. This dye can accumulate when the bacteria membrane is polarized but is released when they are hyperpolarized, resulting in an increase in fluorescence intensity.^36^

Figure 7B displays the fluorescence changes induced by our molecules in *S. aureus* (JE2) over time. Noticeable enhancements in the fluorescence intensity of [DiSC3(5)] were particularly prominent at 2× and 4× MIC for all the drugs tested (Figure S5 shows data for all the concentrations tested). Interestingly, at 4× MIC the compounds showed superior capacity to increase the fluorescence intensity (Figure 7B) when compared to 4× MIC of daptomycin (DAPT, 4 μg/mL), a lipopeptide antibiotic known to gradually dissipate the membrane potential in *S. aureus*.^37^

To further evaluate the effect of the compounds on the bacterial membrane potential we measured the Zeta potential of the *S. aureus* (JE2) (Figure 7C). The Zeta potential is an electrochemical property of cell surfaces reported as net charge.^38^ In general, the net charge on the bacterial surface is negative and is balanced by counterions of opposite charge from the surrounding media.^39^ In this study, the average Zeta potential of untreated *S. aureus* (JE2) was −13.5 mV. A significant alteration in the bacterial surface charge was observed when *S. aureus* (JE2) was exposed to all compounds at 10× MIC. In particular, **5a** showed the highest increase in zeta potential (−3.99 mV), in agreement with the results of the PI uptake and [DiSC3(5)] assays, in which this compound also showed relevant ability to alter the membrane permeability and the membrane potential.

Overall, our findings indicate that our compounds can alter the bacterial membrane potential, which is reflected in the reduction of the zeta potential negative value. These changes in the membrane surface charges might induce the increase in the membrane permeability seen in the PI uptake assay. We hypothesize that changes in membrane potential gradually lead to a discernible yet meaningful shift in membrane permeability observed over time. According to literature reports the use of substances that affect the surface negativity (zeta potential) of membranes can cause the alteration of lipid mediated signaling and membrane destabilization.^40^

To analyze bacterial membrane morphology and to determine the potential of our most compound to produce membrane damage, we performed SEM. As can be observed in Figure 8, the sample treated with **5a**, shows the formation of blebs (indicated by yellow arrows) and roughness on the bacterial surface. In contrast, the untreated control sample (UTD) displays smooth bacterial membrane surfaces. Finally, the results indicate that the compounds cause a change in membrane potential, suggesting an effect on cellular integrity. However, this activity is time and concentration-dependent, indicating that the membrane may not be the main target of action. This effect may contribute to the antibacterial activity of the compounds in a complementary manner, possibly facilitating their entry into the cell and the subsequent modulation of gene expression related to virulence and biofilm formation.

**Figure 8:**
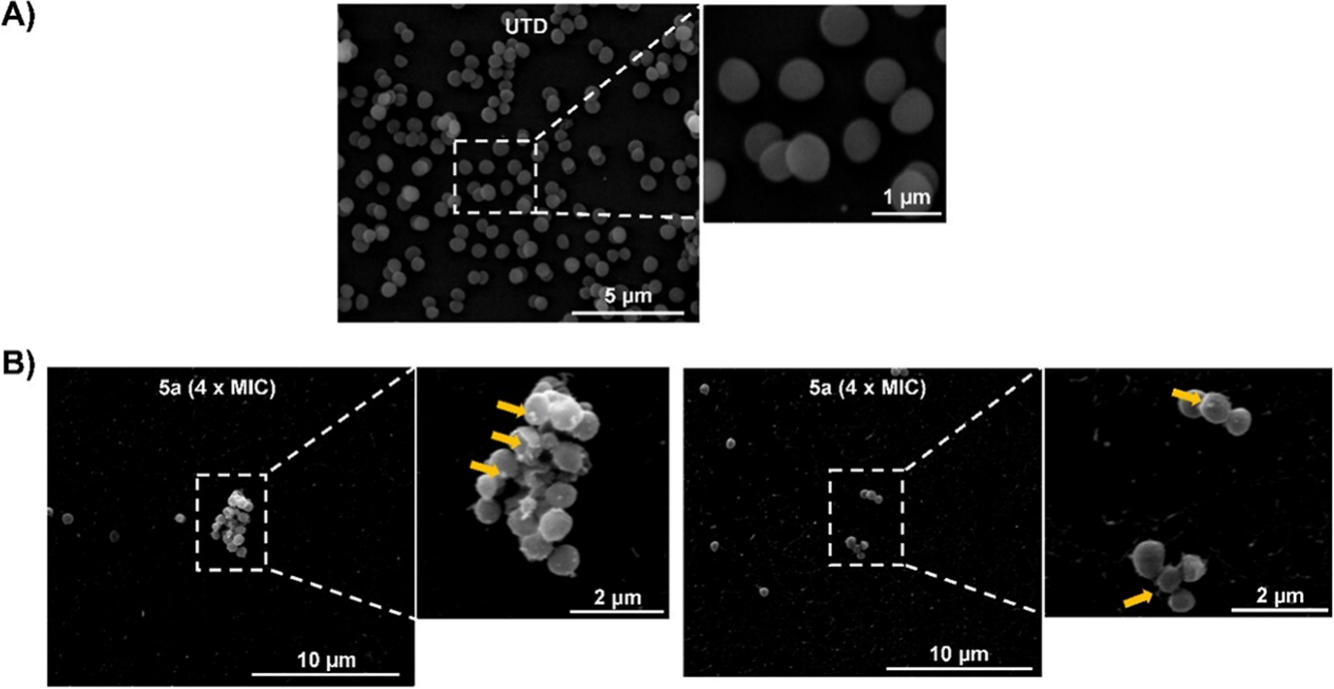
SEM images of UTD control (A) and **5a** at 4× MIC (B) bacteria after 2 h of treatment.

These findings are consistent with the literature on other guanidine-containing compounds, which have been observed to disrupt bacterial membranes. This disruption has been linked to the generation of reactive oxygen species (ROS) and the leakage of intracellular contents.^41^ Although ROS was not directly measured in the present study, the observed membrane depolarization, permeability changes, and extracellular DNA/protein accumulation are consistent with a multifactorial mechanism of cell damage.

#### Antibacterial Activity of Compounds in Combination with Oxacillin

When combined with oxacillin, compound **5a** showed synergistic and additive effects against CA-MRSA and HA-MRSA isolates, respectively (Table 1). The combination of **5a** and the antibiotic reduced the MIC value of the former from 4.68 to 1.17 μg/mL and of the latter from 16 to 2 μg/mL against CA-MRSA. Regarding the HA-MRSA strain, the MIC values were reduced from 4.68 to 1.17 μg/mL for **5a** and from 32 to 16 μg/mL for oxacillin. Compound **5c** also showed an additive effect with oxacillin against community isolates, reducing its MIC from 4.68 to 2.34 μg/mL and oxacillin activity from 16 to 2 μg/mL. No antagonism was observed between any compounds combinations tested.

**Table 1:** Antibacterial Activity of Compounds in Combination with the Antibiotic Oxacillin Against CA-MRSA and HA-MRSA Isolates

	MIC^a^			
	Alone	combined	FICI	result
CA-MRSA				
**5a**	4.68	1.17	0.37	synergistic
oxacillin	16	2		
**5b**	2.34	2.34	1.12	indifferent
oxacillin	16	2		
**5c**	4.68	2.34	0.62	additive
oxacillin	16	2		
HA-MRSA				
**5a**	4.68	1.17	0.75	additive
oxacillin	32	16		
**5b**	2.34	1.17	1.5	indifferent
oxacillin	32	32		
**5c**	4.68	1.17	1.25	indifferent
oxacillin	32	32		

a Concentration in μg/mL; FIC: fractional inhibitory concentration; FICI: fractional inhibitory concentration index.

Considering the potential of compounds **5a**, **5b** and **5c** to act on the bacterial membrane, the synergistic and additive effects in combination with oxacillin may be attributed to the loss of membrane integrity. Potentially facilitated by the positively charged guanidine ligands, as previously suggested for related derivatives, which interact electrostatically with negatively charged bacterial envelopes, increasing intracellular antibiotic accumulation.^42^ These results suggest that our compounds may serve as adjuvants against resistant bacteria, restoring the activity of approved antibiotics like oxacillin, especially in a context of limited antibiotic development and scarce therapeutic options.

#### Compound Stability

A significant challenge in antimicrobial development is stability, as intracellular enzymatic, thermal, hydrolytic, and oxidative mechanisms can degrade drugs. Consequently, we evaluated **5a**, **5b** and **5c** under different conditions related to metabolic processes (Table S2). Table S2 shows that physiological salts (NaCl, CaCl_2_, KCl) increased MIC values, but antimicrobial activity remained promising, with a maximum MIC of 37.44 μg/mL. According to Zhong et al.^43^ the presence of salts can reduce the antimicrobial activity of positively charged molecules by weakening the electrostatic effects with negatively charged membranes.

Compounds were also evaluated for their interaction with trypsin, a digestive protease, as some antimicrobials are protease substrates, limiting use to intravenous or topical routes.^44^ Compound **5c** showed minimal activity loss against *S. aureus* (29213) and HA-MRSA, while **5a** and **5b** maintained their antimicrobial activity in the presence of the hydrolytic enzyme. After absorption, antimicrobials pass through acidic to alkaline environments, making pH stability essential for applicability.^45^ Therefore, compounds were tested under pH conditions, which increased MICs but still allowed inhibition of tested strains at low concentrations (4.68–18.72 μg/mL), indicating minimal impact on activity.^46^

The pharmacokinetics, pharmacodynamics, and drug interactions are influenced by binding to circulating proteins, which can reduce the free active drug fraction.^47^ Accordingly, compounds were evaluated in bovine serum albumin (BSA), fetal bovine serum, blood, and human plasma. In BSA, **5a** remained stable, while **5b** and **5c** showed two- and 4-fold MIC increases, respectively, though not concentration-dependent. The highest MIC (37.44 μg/mL) occurred after 24 h in 50% human serum and plasma. These increases are acceptable if therapeutic levels remain achievable.^48^ Oxacillin MIC also increased.

#### Resistance Development

The initial stages of the discovery of new antimicrobials should assess if the therapeutic candidates induce resistance in microorganisms.^49^ Therefore, the potential for the compounds to induce resistance in *S. aureus* (29213), CA-MRSA and HA-MRSA was evaluated in a 21 day multipass study and oxacillin was used as a control through serial passaging with increasing concentrations of compound (Figure S6).

Compound **5b** did not induce resistance in any tested bacteria. Compound **5a** increased *S. aureus* (29213) MIC from 2.34 to 4.68 μg/mL, while **5c** raised it from 2.34 to 9.37 μg/mL and increased MRSA MICs from 4.68 to 9.37 μg/mL. These MIC increases were minimal compared to oxacillin, which rose 16-fold for *S. aureus* (2 to 32 μg/mL) and 8-fold for CA-MRSA (16 to 128 μg/mL) and HA-MRSA (32 to 256 μg/mL).

As evidenced in mechanism assays, our compounds target the cell membrane, possibly affecting other sites. Like polymyxins and daptomycin, which act on conserved membranes, they have low resistance potential since altering membrane composition is energetically costly and risky for microorganism.^50^ Thus, these compounds are characterized as low-resistance inducers with safe therapeutic potential in relation to the emergence of resistance.

#### Biocompatibility of Compounds

Next, we evaluated the mutagenic, hemolytic and cytotoxic potential of the compounds in vitro, in addition to evaluating their toxicity in a *T. molitor* larvae in vivo model. The ICH S2 (R1) guidelines recommend genotoxicity assays, including the bacterial gene mutation test (Ames), widely used in regulatory screening due to its high sensitivity and predictive value for carcinogenicity.^51,52^ Accordingly, the in vitro genetic safety of the compounds was evaluated through the Ames test, using *Salmonella typhimurium* strains TA98, TA100 and TA102 and employing exogenous metabolism, the S9 fraction.

The results of the Ames test with the compounds are shown in Table S3. We observed the molecules did not demonstrate mutagenic potential. Although some evaluated concentrations significantly increased the number of revertant colonies, no mutagenicity index greater than two was observed, nor was a dose–response relationship against strains of *S. typhimurium* TA98, TA100 and TA102 with and without metabolic activation. These results indicate that, under these conditions, compounds do not have direct and indirect potential to cause mutations such as frameshift and base pair substitution. Similar findings have been reported for related cationic compounds, such as cyanoguanidine derivatives complexed with metals and PHMB (commercially registered as Monogin), which were also considered nonmutagenic in Ames assays and safe for therapeutic use.^53,54^

Regardless of administration route, antimicrobials enter the bloodstream and interact with components mainly composed of red blood cells; thus, hemolysis testing is widely used to assess toxicity.^55^ The compounds were tested at a range of concentrations (from 1 to 1024 μg/mL) to determine the concentrations that caused 10% and 50% hemolysis, as indicated in Table S4. The results indicate that the compounds are not hemolytic, especially when considering the MIC value.

Subsequently, **5a**, **5b** and **5c** were evaluated against two human cell lines to verify if the compounds alter functions or induces cell death. HEK-293 cells displayed high viability in the presence of the studied molecules (around 90% survival in the evaluated concentration range for all compounds (Figure S7B). HEp-2 cells showed a more sensitive profile against the tested molecules, at the highest tested concentration (128 μg/mL), around 70% of cell viability was seen (Figure S7A). In general, the compounds did not show cytotoxic potential for HEK-293 and HEp-2 cell lines, maintaining acceptable viability, especially when considering their MIC values. These results indicating a good potential of our compounds to become drug candidates by combining high antimicrobial potency (low MIC), blood compatibility, and lack of cytotoxic and mutagenic effects.

Finally, to gain further insights into the systemic toxicity of the tested compounds, we employed an in vivo survival assay using *T. molitor*, a well-established model for preliminary toxicological evaluation of bioactive molecules.^56^ Treatment with compound **5c** resulted in a significant increase in the survival rate of *T. molitor* larvae infected with *S. aureus* (JE2), compared to the positive control group (Figure 9A,B). All tested concentrations (1×, 2×, and 4× MIC) exhibited statistically significant protective effects relative to the positive control, with survival outcomes comparable to those observed in the PBS and vancomycin-treated groups. Notably, the 4× MIC concentration of **5c** led to a 90% survival rate over 168 h, matching that achieved with vancomycin (2 μg/mL), the reference antibiotic for MRSA infections. The 2× and 1× MIC concentrations also maintained protective effects, with survival rates exceeding 80% and 70%, respectively, reinforcing the compound’s efficacy even at lower doses. These findings suggest that **5c** may help to limit the progression of systemic *S. aureus* infection, potentially by modulating the host immune response to the bacterial challenge.

**Figure 9:**
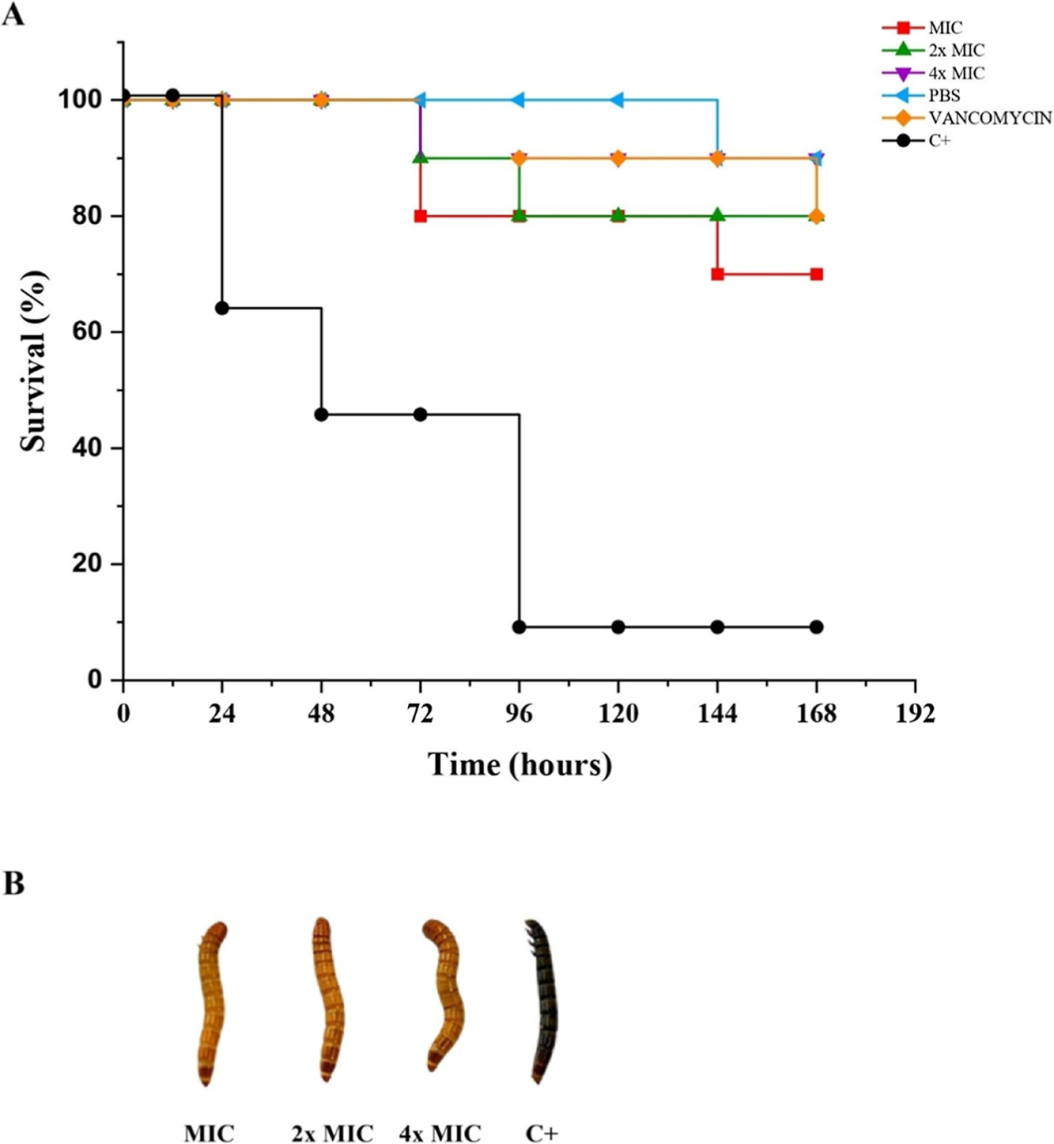
Survival of *T. molitor* larvae infected with *S. aureus* JE2 and treated with compound **5c**. (A) Survival curves over 168 h postinfection; treatments: PBS (negative control), vancomycin (2 μg/mL), compound **5c** at 1×, 2×, and 4× MIC; positive control untreated. (B) Representative image showing live (light, active) and dead (dark, immobile) larvae. *N* = 10/group. Log-rank test: all treatments significantly improved survival vs positive control (*P* < 0.001).

## Conclusion

Antimicrobial resistance remains one of the major public health threats worldwide, highlighting the urgent need for new therapeutic alternatives. In this study, three guanidine-functionalized compounds derived from 3,5-diaryldene-4-piperidone were synthesized and showed potent antibacterial activity against *S. aureus*, including MRSA, with low MIC values and a bacteriostatic profile. Beyond activity against planktonic cells, the compounds demonstrated antibiofilm potential by significantly reducing biomass and metabolic activity, altering cell morphology, and compromising membrane integrity as evidenced by multiple analyses. Modulation of gene expression related to virulence, biofilm formation, and matrix structure was also observed, suggesting a multifactorial mechanism of action for these molecules. The compounds affected bacterial membranes by altering permeability and membrane potential, associated with changes in zeta potential and structural damage visualized by confocal microscopy. Antibacterial activity was enhanced when combined with oxacillin, supporting their potential use in adjuvant therapy. In an ex vivo porcine skin model, the antibiofilm efficacy was maintained, reinforcing their applicability in biologically complex environments. Additionally, the compounds exhibited stability under various physiological conditions and showed low resistance induction potential following prolonged exposure. Biocompatibility was supported by negative results in mutagenicity, hemolysis and cytotoxicity in vitro and in vivo efficacy was also demonstrated through increased survival in an infected animal model. Overall, the compounds developed in this study emerge as promising candidates for the development of novel antimicrobial agents, combining multiple mechanisms of action, antibiofilm activity, stability, low resistance potential, and biocompatibility.

## Materials and Methods

### Design and Synthesis

Compounds **5a**–**5c** were synthesized via adapted literature procedures involving aldol condensation and subsequent cyclization with guanidine hydrochloride. Reactions were monitored by TLC and purified by flash chromatography. Purity (>95%) was confirmed by HPLC. Structures were verified by ^1^H, ^13^C, and ^19^F NMR spectroscopy and high-resolution mass spectrometry (HRMS). Detailed synthetic procedures, compound characterization data, and spectral analyses are provided in the Supporting Information.

### Biological Evaluation

Microorganisms were obtained from ATCC (Rockville, MD, USA), including Gram-positive *S. aureus* (29213), *Bacillus cereus* (11778), *Staphylococcus saprophyticus* (BAA750), and Gram-negative *S. typhimurium* (14028), *Klebsiella pneumoniae* (700603). Two MRSA clinical isolates (CA-MRSA and HA-MRSA), carrying the *mecA* gene, were sourced from the Laboratório de Microbiologia Aplicada (UFGD). Resistance profiles were confirmed by Vitek2 (BioMérieux) and *mec*A detection by PCR (Table S5). *S. aureus* USA300 JE2 was kindly provided by Dr. Kenneth Bayles (Department of Pathology and Microbiology, UNMC).

### Antimicrobial Susceptibility Tests

The minimum inhibitory concentration and the minimum bactericidal concentration were determined by the broth microdilution method according to the guidelines established by the Clinical and Laboratory Standards Institute with some adaptations.^57^ MIC was determined using 96-well microplates with 100 μL of MHB and test compounds, serially diluted to 300–0.58 μg/mL. Then, 10 μL of a bacterial suspension (10^6^ CFU/mL) was added. Plates were incubated at 37 °C for 24 h, followed by the addition of 0.1% triphenyl tetrazolium chloride to assess bacterial respiration. MIC was defined as the lowest concentration without visible growth. For MBC, aliquots from MIC wells showing no growth were plated on MHA and incubated at 37 °C for 24 h; the lowest concentration yielding no CFUs was recorded as the MBC. Ampicillin was used as the reference antibiotic, while oxacillin was employed for MRSA strains. The corresponding MIC values are provided in Table S1.

### Growth Kinetics Study

The growth kinetics study followed a protocol that was based on the one established by Klepser et al.^58^ with some modifications. Falcon tubes containing MHB and bacterial strains *S. aureus* (29213) and HA-MRSA standardized to 1 × 10^8^ cells/mL and diluted 1:100 were supplemented with concentrations equivalent to 4×, 2×, and 1× MIC of **5a**, **5b**, and **5c**. Positive and negative controls consisted of untreated cells and cells treated with oxacillin, respectively. The tubes were incubated at 37 °C and 70 rpm. At intervals of 0, 2, 4, 6, 8, 10, 12, and 24 h, a 100 μL aliquot of each sample was serially diluted and plated on MHA. The plates were then incubated for 18–24 h, and viable cells were counted, and the results were converted into Log_10_CFU.

### Antibiofilm Activity

The antibiofilm potential of **5a**, **5b** and **5c** was determined according to the methodology proposed by Costa et al.^59^ with adaptations. Antibiofilm concentrations were 1×, 2×, and 4× MIC, based on susceptibility results. Antibiofilm activity was evaluated using *S. aureus* (29213) and two MRSA clinical isolates (CA-MRSA and HA-MRSA). Strains were grown in TSB (24 h, 37 °C, 70 rpm), washed with PBS, centrifuged (3500 rpm, 3×), and resuspended in TSB to 1 × 10^8^ cells/mL.

To assess biofilm inhibition, compounds were diluted in TSB +1% glucose, and 196 μL was added to 96-well plates with 4 μL of bacterial suspension. Plates were incubated (37 °C, 48 h, 70 rpm), then washed with PBS (3×). Positive (TSB +1% glucose and bacteria) and negative (TSB +1% glucose only) controls were included. Triplicates were used. For biofilm disruption, 196 μL of TSB +1% glucose and 4 μL of bacterial suspension were added to 96-well plates and incubated (37 °C, 48 h, 70 rpm). Wells were washed (PBS, 3×), then **5a**, **5b**, and **5c** were added at defined concentrations and incubated (37 °C, 24 h, 70 rpm). Wells were washed again to remove nonadherent cells. Positive (TSB +1% glucose and bacteria) and negative (TSB +1% glucose only) controls were included. Triplicates were used.

### Biofilm Characterization

The antibiofilm potential of the compounds from previous described assays were evaluated in terms of: (1) Metabolic activity, mediated by colorimetric detection of viable cells using the reagent XTT proposed by Pierce et al.^60^ with some modifications; (2) Biomass, using the CV staining proposed by Silva et al.^61^ with some modifications; (3) Scanning electron microscopy proposed by Ramalho et al.^62^ and (4) fluorescence microscopy stained with the Filmtracer LIVE/DEAD Biofilm Viability Kit from Invitrogen. In methods (1) and (2) the percentage of biofilm inhibition was calculated according to the equation: % Inhibition = (positive control absorbance – sample absorbance)/positive control absorbance x 100.

### Antibiofilm Activity in an Ex Vivo Model of the Porcine Skin

The antibiofilm activity of **5c** was assessed using an ex vivo porcine skin model, adapted from Horton et al.^63^ Skin samples (12 mm) were decontaminated in streptomycin (1000 μg/mL) and penicillin (1000 U/mL) for 18 h, washed with DPBS, and placed in 12-well plates containing semisolid DMEM with 10% FBS. Suspensions of *S. aureus* (29213), CA-MRSA, and HA-MRSA (1 × 10^6^ cells/mL) were applied (20 μL), followed by 20 μL of **5c** at 2× or 4× MIC. After 24 h at 37 °C, samples were transferred to NaCl, vortexed (10 min), and plated on Mannitol agar. CFUs were counted after 24 h incubation at 37 °C. Biofilm inhibition (%) was calculated as [(CFU/mL control – CFU/mL treatment)/CFU/mL control] × 100.

### Expression of Genes Using Quantitative Real-Time PCR

RNA from *S. aureus* JE2 24 h biofilms was isolated following exposure to 2×, 3×, and 4× MIC of **5a**, vancomycin, or BHI +10% glucose (control). RNA extraction was performed using the SV Total RNA Isolation System (Promega, WI, USA). RT-qPCR reactions (20 μL) included 0.4 μL OneStep Mix, 10 μL GoTaq Mix, 10 mmol of each primer, and 20 ng RNA as described by Humeniuk et al.^64^ Virulence gene expression was quantified using the OneStep Real-Time PCR System (Applied Biosystems, MA, USA) under manufacturer-recommended cycling conditions. 16S rRNA served as the internal control. Each assay included triplicates, a vancomycin-treated positive control, and an untreated negative control. Relative expression was calculated via the 2^–ΔΔ*Ct*^ method.^64^

### Structural Characterization of the Biofilm Matrix by Confocal Laser Scanning Microscopy

Structural analysis of the biofilm matrix was performed following gene expression studies to correlate molecular changes with biofilm composition. To characterize the biofilm structure, sample preparation was carried out following the methods proposed by Scaffo et al.^65^ with modifications. Specifically, TOTO-1 was used at a final concentration of 2 μM (Ex: 514/Em: 533), Wheat Germ Agglutinin Alexa Fluor 647 at 5 μg/mL (Ex: 650/Em: 665) (Invitrogen, MA, USA), and 200 μL of SYPRO Ruby Red (Ex: 408/Em: 640). Dyes were applied directly in the SYPRO Ruby Red solution. A custom CellProfiler pipeline was implemented to quantify the fluorescent intensity of each cell as well as the area outside the cells.

### Propidium Iodide Uptake

Inner membrane damage was assessed via the PI uptake assay.^66^ *S. aureus* JE2 was grown on MHA at 37 °C to mid log phase, then resuspended in HEPES buffer (5 mM, pH 7, with 3.6 μg/mL glucose) at 0.3 McFarland. Compounds **5a**, **5b**, and **5c** were prepared in PBS at 5× the target concentration to yield 1×, 2×, and 4× MIC. In 96-well plates, 40 μL of compound solution, 150 μL of bacterial suspension, and 10 μL of PI (6.7 μg/mL) were combined (final volume: 200 μL). Plates were incubated in the dark at 37 °C for 30 min to 6 h. PI-stained bacterial suspension served as untreated control; HEPES + PI was the negative control. For daptomycin, HEPES was supplemented with 50 μg/mL CaCl_2_. Fluorescence (excitation 535 nm, emission 615 nm) was measured using a SpectraMax reader (Molecular Devices).

### Voltage-Sensitive Dye 3,3′-Dipropylthiadicarbocyanine Iodide [DiSC3(5)] Assay

Alterations in the bacterial membrane potential were evaluated by voltage-sensitive [DiSC3(5)] assay.^36,66^ *S. aureus* JE2 was grown on MHA (37 °C) to mid log phase, then adjusted to 0.5 McFarland in HEPES buffer (5 mM, pH 7, with 3.6 μg/mL glucose). Compounds **5a**–**5c** were prepared in PBS at 5 times the concentration to obtain 1×, 2×, and 4× MIC. In 96-well plates, 40 μL of each compound and 158 μL of bacterial suspension were added and incubated in the dark (37 °C, 30 min). Next, 2 μL of [DiSC3(5)] (diluted in DMSO) were added into the wells to reach a concentration of 1 μM. Further, a final concentration of 1% DiSC3(5)-DMSO was used to maintain appropriate dye solubility. Plates were further incubated (37 °C, 30 min–6 h). Untreated and buffer-only controls were included. For daptomycin, buffer was supplemented with 50 μg/mL CaCl_2_. Fluorescence (Ex 622 nm, Em 670 nm) was measured with a SpectraMax reader (Molecular Devices).

### Bacterial Surface Zeta Potential Determination Assay

Changes in the bacterial surface potential were evaluated by the measurement of the bacterial zeta potential.^40^ *S. aureus* JE2 was seeded in MHA and incubated at 37 °C to reach mid log phase. Next, a bacterial cell suspension of 0.5 McFarland was prepared in PBS. **5a**, **5b** and **5c** were prepared in PBS at 2× the concentration to obtain 1×, 2× and 4× MIC (MIC = 2 μg/mL). Following, 750 μL of the prepared bacterial suspension was mixed with 750 μL of the studied compound solutions. The final solutions were incubated for 30 min at 37 °C. The bacterial Zeta potential was measured using a Zeta sizer Nano ZS90 device (Malvern instruments), with attenuation fixed to 6 and at room temperature (28 °C). Each experiment was carried out under identical experimental conditions (50 scans maximum for 3 runs).

### Evaluation of Bacterial Membrane Morphology after Treatment by Scanning Electron Microscopy

MRSA suspensions (10 mL, 10^8^ cells/mL) were treated with **5a** (4× MIC) for 2 h at 37 °C with shaking (75 rpm). Cells were washed with saline (10 mL) and fixed in 2% glutaraldehyde and 2% paraformaldehyde in 0.1 M Sorenson’s phosphate buffer (pH 6.2) at 4 °C for at least 24 h. Samples were washed thrice with phosphate buffer, postfixed in 1% osmium tetroxide for 30 min, and dehydrated through an ethanol series (50%, 70%, 90%, 95%, 100% ×3). After critical point drying, samples were mounted on aluminum stubs with carbon tape, coated with silver paste for conductivity, sputter-coated with ∼50 nm gold–palladium alloy, and imaged at 30 kV in a FEI Quanta 200 SEM under high vacuum.

### Assessment of Activity Potential when Combined with Oxacillin

The synergism assay was performed according to De Castro et al.^67^ To determine the combinatorial action, concentrations of **5a**, **5b** and **5c** ranging from 37.50 to 0.29 μg/mL and of the antibiotic oxacillin concentrations from 128 to 0.5 μg/mL were tested. In a 96-well microplate, a combined matrix of compounds and the antibiotic in different concentrations, along with the bacterial strains were incubated at 37 °C for 24 h. For result interpretation, the Fractional Inhibitory Concentration Index was calculated: (FICI) = FIC_compounds_ (combination compounds MIC/compounds alone MIC) + FIC_antibiotic_ (combination oxacillin MIC/oxacillin alone MIC). Results are interpreted as FICI ≤0.5 Synergism; Additive effect (0.5 < FICI <1); Indifferent effect (1 ≤ FICI <4) and antagonistic effect FICI ≥ 4.^68^

### Stability of Compounds

To verify the stability, **5a**, **5b** and **5c** were treated in different conditions of salts, plasma, serum, pH and hydrolytic enzyme and incubated for 4 h at 37 °C.^69^ After incubation, the minimum inhibitory concentration of compounds against *S. aureus* (29213), CA-MRSA and HA-MRSA were determined.^57^ Ethical approval was granted by the committees of UFGD and Plataforma Brasil (Opinion No. 5,588,196).

### Resistance Development

The multistep resistance study was designed to verify whether **5a**, **5b** and **5c** were able to induce resistance in *S. aureus* (29213), CA-MRSA and HA-MRSA. For this, the bacterial suspensions were exposed to two dilutions below the MIC of the compounds and oxacillin for 21 days. At each subsequent passage, the broth microdilution assay was employed to determine weather there had been a change in the MIC value.^57,70^

### Biocompatibility of Compounds

#### Ames Test

The Ames test was performed following the microsuspension method by Kado et al.^71^ and OECD Guideline 471,^72^ using *S. typhimurium* strains TA97a, TA98, TA100, and TA102, standardized to 10^8^ cells/mL. Assays were conducted with and without metabolic activation (S9). Each tube received 50 μL of phosphate buffer or S9 mix, 5 μL of compounds **5a**, **5b**, or **5c** (at 5000, 1500, 500, 150, and 50 μg/plate), and 50 μL of bacterial suspension. After 90 min preincubation at 37 °C, 2 mL of top agar were added and the mixture poured onto minimal agar plates. Plates were incubated at 37 °C for 48–66 h, and revertant colonies were counted. All assays were performed in triplicate.

#### Hemolytic Activity

The potential of our compounds to lyse erythrocytes was evaluated using human blood from healthy donors according to the methodology proposed by Dhonnar et al.^73^ with some modifications. Ethical approval was granted by the committees of UFGD and Plataforma Brasil (Opinion No. 5,588,196).

Five mL of human blood from healthy donors were collected in EDTA tubes, homogenized, and centrifuged (10 min, 1600 rpm, 20 °C). The pellet was washed with PBS and centrifuged (3×). It was then resuspended in PBS to obtain a 1% erythrocyte solution. Samples were mixed 1:1 with the 1% solution. Triton X-100 (0.01%) and PBS served as positive and negative controls, respectively. Tubes were incubated (37 °C, 1 h, slow agitation), centrifuged, and the supernatant read at 540 nm. Triplicates were used. % Hemolysis = (*A*_540_sample – *A*_540_negative)/(*A*_540_triton – A_540_negative) × 100.

#### Cytotoxicity Assay

HEK-293 cells were cultured in DMEM (Gibco 11965–092) with 10% FBS, 1% penicillin/streptomycin (Gibco 15140–122), and 200 mmol l-glutamine at 37 °C in 5% CO_2_. Human epithelial cells were grown in DMEM with 10% FBS and 10 μg/mL gentamicin under the same conditions. After at least passages (70–100% confluence), cells were seeded at 5 × 10^3^/well in 96-well plates, incubated 24 h, and treated with compounds (128–8 μg/mL, in triplicate). DMSO was used as vehicle control. Plates were incubated for another 20 h, then washed twice with PBS and replenished with fresh media. XTT solution (50 μL; final well concentrations: 400 μg/mL XTT, 50 μg/mL menadione) was added and plates incubated for 4 h. Absorbance was read at 450 nm using a Multiscan FC photometer (Thermo Fisher).

#### In Vivo Survival Assessment in *T. molitor* Larvae Model

The in vivo effect of compound **5c** on *T. molitor* larvae infected with *S. aureus* (JE2) was evaluated using a survival assay adapted from Morey et al.^74^ Larvae (100–200 mg) with uniform coloration and no signs of melanization or lesions were injected with 5 μL of a standardized bacterial suspension into the hemocoel via the second ventral sternite using a Hamilton syringe (10 μL, 26G). One hour postinfection, treatment groups received sterile PBS (negative control), vancomycin (2 μg/mL), or **5c** at 1×, 2×, or 4× MIC; an untreated infected group served as a positive control. Larvae were incubated at 37 °C, and survival was assessed every 12 h for 168 h based on response to mechanical stimulation and melanization.

### Statistical Analysis

Statistical analyses were performed using GraphPad Prism 9.0.0 (GraphPad Software, CA, USA). Comparisons between two groups used Student’s *t*-test; one-way ANOVA with Dunnett’s post hoc and two-way ANOVA with Šídák’s test were applied as appropriate (*p* < 0.05). Larval survival was analyzed using the Log-rank (Mantel–Cox) test. Ames test data were evaluated with Salanal software (EPA, USA, v1.0) per Bernstein et al.^75^ Graphs were generated using GraphPad Prism and Origin 2021 (OriginLab, MA, USA). Differences were considered significant at *p* ≤ 0.05.

## Supplementary Material



## Notes

The Supporting Information includes materials and methods—design and synthesis (Section 1), MIC data against Gram-positive and Gram-negative strains (Table S1), MICs under different conditions (Table S2), mutagenicity results (Table S3), hemolytic activity (Table S4), and resistance profiles via Vitek2 (Table S5). It also presents bacterial growth kinetics (Figure S1), biofilm inhibition and eradication (Figure S2), biofilm cell viability (Figure S3), membrane permeabilization and potential analyses (Figures S4–S5), resistance development over 21 days (Figure S6), cytotoxicity in HEp-2 and HEK-293 cells (Figure S7), and compound characterization by NMR, HPLC, and HRMS (Section 13) (PDF)

## Abbreviations USED

ANOVA: analysis of variance
CFU: colony forming units
Cont: control
CV: crystal violet
DAPT: daptomycin
DMEM: Dulbecco’s modified Eagle medium
DMSO: dimethyl sulfoxide
DPBS: Dulbecco’s phosphate saline buffer
FBS: fetal bovine serum
FIC: fractional inhibitory concentration
FICI: fractional inhibitory concentration index
HEK-293: immortalized human embryonic kidney cell line
HEp-2: epithelial type 2 cell line
HP: human plasm
ICH: International Council for Harmonization of Technical Requirements for Pharmaceuticals for Human Use
MBC: minimum bactericidal concentration
MHA: Müeller–Hinton agar
MHB: Müeller–Hinton broth
MI: mutagenic index
MIC: mininimum inhibitory concentration
OECD: Organization for Economic Cooperation and Development
Oxa: oxacillin
PBS: phosphate buffered saline
PHMB: polyhexamethylene biguanide
PI: propidium iodide
SCC*mec*: Staphylococcal chromosomal cassette
SH: human serum
TRIP: tripsin
TSB: trypticase soy broth
UTD: untreated
WHO: World Health Organization
XTT: (2,3-bis­(2-methoxy-4-nitro-5-sulfophenyl)-5-[(phenylamino)­carbonyl]-2*H*-tetrazolium hydroxide)
