# Supplementary material for: Biocompatible Guanidine-Functionalized Compounds with Biofilm and Membrane Disruptive Activity Against MRSA
Source: ACS Infect Dis. 2025 Sep 15;11(10):2907–23. doi: 10.1021/acsinfecdis.5c00642 (PMC12519469; doi:10.1021/acsinfecdis.5c00642)
Supplement: Supplementary file 1 [file id5c00642_si_001.pdf]

Supporting information

Biocompatible guanidine-functionalized compounds with biofilm and membrane disruptive activity against MRSA

Pamella Fukuda de Castilho[a], Luana Janaína de Campos[b], Audifás-Salvador Matus-Meza[b], Huihua Xing[b], Diana Liz Jimenez Rolão[a], Fernanda Galvão[c], Fabiana Gomes da Silva Dantas[c], Rongguo Ren[d], Cameron Dobrotka[e], Fabio Aguiar-Alves[e,f], Martin Conda-Sheridan[b*], Kelly Mari Pires de Oliveira[c*]

[a]Faculty of Health Sciences, Federal University of Grande Dourados, Dourados, MS, 79804-970, Brazil.
[b]Department of Pharmaceutical Sciences, College of Pharmacy, University of Nebraska Medical Center, Omaha, Nebraska, 68198, United States.
[c]Faculty of Biological and Environmental Sciences, Federal University of Grande Dourados, Dourados, MS, 79804-970, Brazil.
[d]College of Pharmacy, University of Nebraska Medical Center, 986125 Nebraska Medical Center, Omaha, Nebraska, 68198-6125, United States.
[e]Department of Pharmaceutical Sciences, Lloyd L. Gregory School of Pharmacy, Palm Beach Atlantic University, West Palm Beach, Florida, 33401, United States.
[f]Postgraduate Program in Applied Microbiology and Parasitology and Postgraduate Program in Pathology, Fluminense Federal University, Niteroi, RJ, 24220-900, Brazil.

* Corresponding authors: Kelly Mari Pires de Oliveira (kellyoliveira@ufgd.edu.br) and Martin Conda-Sheridan (martin.condasheridan@unmc.edu)

Table of Contents

1. **Materials and Methods** – Design and synthesis of compounds.............................. S4-S6

2. ^{1}H NMR, ^{13}C NMR, HPLC and HRMS Spectra of the synthesized compounds........... S7-S21

3.**Table S1.** Minimum Inhibitory Concentration (µg.mL^{-1}) of compounds against Gram-positive and Gram-negative strains.. S22

4.**Table S2.** Minimum inhibitory concentration (µg/mL) of compounds after treatment with different conditions.. S23-S24

5.**Table S3**. Mutagenic activity expressed by mean revertants/plate ± standard deviation of compounds against strains TA98, TA100, TA102 of *S.* Typhimurium with metabolic activation (S+) and without metabolic activation (S-)... S25

6.**Table S4.** Hemolytic activity of the compounds.. S26

7.**Table S5.** Resistance profile of the isolates by susceptibility testing using the automated Vitek®2 system... S26

8.**Figure S1.** Effect of compounds on bacterial growth kinetics. The bacteria at a concentration of 1x10^{8} cells/mL and diluted 1:100 were initially inoculated in culture medium with concentrations equivalent to 4x, 2x and 1x MIC of compounds. Time points were recorded at 0, 2, 4, 6, 8, 10, 12 and 24 hours after inoculation. Data represent the mean ± standard deviation (N = 3). Omitted error bars indicate an SD value shorter than the size of the symbol presented in the plots... S27

9.**Figure S2.** Potential of the compounds to inhibit (**A**, **B** and **C**) and to destroy (**D**, **E** and **F**) *S. aureus* (ATCC 29213), CA-MRSA and HA-MRSA biofilms evaluated according to biomass.. S28

10.**Figure S3.** Cell viability of HA-MRSA biofilms by live/dead staining and fluorescence microscopy after treatments with compounds in the biofilm formation inhibition assay. The biofilms were cultivated in the presence of different concentrations of the compounds from an initial inoculum of 1 x 10^8 cells/mL for 48 hours. Living cells are stained fluorescent green and dead cells fluorescent red. **(A)** Control; **(B)** Treated at 1x MIC concentration of compound **5a**; **(C)** Treated at 1x MIC concentration of compound **5b**; **(D)** Treated at 1x MIC concentration of compound **5c.**.. S29

11. **Figure S4.** Inner membrane permeabilization evaluation by PI uptake against *S. aureus* JE2 displaying statistical analyses. Error bars report SEM. Each experiment included 3 biological replicates. $P \leq 0.0001$; ns: not significant by One-Way ANOVA with Dunnett's multiple comparations test………………………………………………………………. S30

12.**Figure S5.** Kinect membrane activity investigation for the studied compounds at different concentrations against *S. aureus* JE2. **(A)** Inner membrane permeabilization evaluation by PI uptake; **(B)** Membrane potential analysis by the voltage sensitive fluorescent dye [DiSC3(5)]……………………………………………………….... S30

13.**Figure S6.** Study of resistance development in 21 days. The bacterial strains were cultivated in MHB at a concentration of ¼ MIC of the compounds. The broth microdilution assay was employed to determine the MIC value after each passage during the 21-day period………………………………………………………………………... S31

14. **Figure S7.** Viability of **5a**, **5b** and **5c** against HEp-2 **(A)**, HEK-293 **(B)** cell lines. Error bars report SEM (N = 3). Each experiment included technical replicates. Omitted error bars indicate an SD value shorter than the size of the symbol presented in the plots……….. S31

MATERIALS AND METHODS

Design and synthesis

All chemicals and starting materials were obtained from Fisher Scientific, ACROS Organics, TCI, and Alfa Aesar and used as received. The reactions were monitored by thin layer chromatography (TLC) using a UV lamp (analyzed at 254 and 365 nm) and some reactions were revealed by iodine chamber. The purification of the compounds was done by flash chromatography (silica gel, pore size 60 Å, 230- 400 mesh).

The products were identified by ^{1}H NMR, ^{13}C NMR and ^{19}F NMR using a Bruker 500 MHz spectrometer. All NMR samples were dissolved in $CHCl_3$, D_2O, DMSO-d_6, or CD_3OD and were referenced using the deuterated solvent itself. High-Resolution Mass Spectrometry (HRMS) analyses were obtained at the University of Nebraska Medical Center core facility, using electrospray ionization (ESI) techniques.

The purity of all compounds was over > 95% and determined on an Agilent 1200 HPLC system under a wavelength of 254 nm and using Kinetex 5 um EVO C18 100 Å; LC Column 250 X 4.6 mm; P/No.: 00G-4633-E0; S/No.: H19-120120; B/No.: 5720-0090. The Compounds were named using the naming algorithm employed by ChemDraw Professional vers. 15.0 (Perkin Elmer).

General procedure for the synthesis of 1-benzyl-3,5-di((E)-benzylidene)piperidin-4-one derivatives (a). An aldol condensation was performed to obtain the 3,5-diaryldene-4-piperidone intermediates (**3a-c**, Scheme 1) by adapting the procedure reported by Anthwal et al.[19] To a mixture of 1-benzylpiperidin-4-one (5 mmol) in EtOH (20 mL) were added a desired carboxaldehyde aromatic derivative (10.25 mmol) and 5 mL of aqueous NaOH (1 M, 5 mmol). The reaction mixture was stirred at room temperature for 6 h. After completion of the reaction, the reaction mixture was neutralized with HCl (0.1 M) to afford a solid product. Following, the precipitate was filtered and the solid was washed with EtOH/H_2O 1:1 to give the desired product.

General procedure for the synthesis of 3,4-dihydropyrimidin-2(1H)-imine (b). To obtain the final products (**5a-c**, Scheme 1), the corresponding 3,5-di((*E*)-benzylidene)piperidin-4-one intermediates (**3a-c,** Scheme 1, 0.72 mmol) and guanidine hydrochloride (2.16 mmol) were dissolved in 20 mL of isopropanol. This solution was stirred for 10 minutes, then aqueous NaOH (4M, 1.5 mL) was added, and the reaction continued at 90–100 °C for 6h. After, the solution was neutralized with HCl (1M) and the

product was extracted with EtOAc (3 x 40 mL). The combined organic layer was dried over Na_2SO_4 and was concentrated by rotary evaporation. Finally, the product was purified by flash chromatography to give a yellow solid.

Characterization of the final compounds

*(E)-6-benzyl-8-(4-chlorobenzylidene)-4-(4-chlorophenyl)-3,4,5,6,7,8-hexahydropyrido[4,3-d]pyrimidin-2(1H)-imine (**5a**).* Yellow solid, yield: 65%. ^{1}H NMR (500 MHz, Chloroform-*d*) δ 2.65 (d, *J* = 16.4 Hz, 1H), 2.93 (d, *J* = 16.5 Hz, 1H), 3.35 (d, *J* = 14.0 Hz, 1H), 3.41 – 3.53 (m, 2H), 3.61 (d, *J* = 14.0 Hz, 1H), 4.80 (s, 1H), 7.01 (d, *J* = 8.2 Hz, 2H), 7.07 (s, 1H), 7.08 – 7.12 (m, 4H), 7.16 (d, *J* = 8.1 Hz, 2H), 7.20 – 7.23 (m, 3H), 7.28 (d, *J* = 8.1 Hz, 2H), 7.73 (s, 2H), 8.87 (s, 1H), 9.96 (s, 1H). ^{13}C NMR (125 MHz, Chloroform-*d*) δ = 51.59, 52.16, 55.98, 61.03, 111.00, 123.79, 125.55, 126.22, 127.58, 128.47, 128.48, 128.57, 129.05, 129.60, 130.60, 133.38, 134.27, 135.01, 137.11, 138.45, 152.28. HPLC purity, 95.78. HRMS (m/z): $[M+H]^+$ calcd for $C_{27}H_{24}Cl_2N_4$, 475.1456; found, 475.1045.

*(E)-6-benzyl-8-(2-methylbenzylidene)-4-(o-tolyl)-3,4,5,6,7,8-hexahydropyrido[4,3-d]pyrimidin-2(1H)-imine (**5b**).* Yellow solid, yield: 88%. ^{1}H NMR (500 MHz, Chloroform-*d*) δ 2.26 (s, 3H), 2.28 (s, 3H), 2.64 (d, *J* = 16.4 Hz, 1H), 2.90 (d, *J* = 16.6 Hz, 1H), 3.30 (d, *J* = 13.9 Hz, 1H), 3.45 (m, 2H), 3.59 (d, *J* = 13.9 Hz, 1H), 5.04 (s, 1H), 6.92 (d, *J* = 7.6 Hz, 1H), 7.02 (dt, *J* = 8.6, 4.2 Hz, 1H), 7.07 (d, *J* = 7.4 Hz, 1H), 7.11 (m, 4H), 7.16 (dd, *J* = 6.2, 2.7 Hz, 1H), 7.20 (m, 6H), 7.59 (brs, 1H), 8.62 (s, 1H), 9.92 (s, 1H). ^{13}C NMR (125 MHz, Chloroform-*d*) δ = 19.17, 20.36, 51.81, 52.36, 53.20, 60.88, 110.22, 124.20, 125.31, 125.53, 126.05, 127.15, 127.40, 127.60, 128.03, 128.32, 128.87, 129.04, 129.15, 130.07, 131.37, 135.01, 135.83, 137.12, 137.29, 137.65, 152.19. HPLC purity, 98.33. HRMS (m/z): $[M+H]^+$ calcd for $C_{29}H_{30}N_4$, 435.2548; found, 435.2601.

*(E)-6-benzyl-8-(4-fluorobenzylidene)-4-(4-fluorophenyl)-3,4,5,6,7,8-hexahydropyrido[4,3-d]pyrimidin-2(1H)-imine (**5c**).* Yellow solid, yield: 80%. ^{1}H NMR (500 MHz, Chloroform-*d*) δ 2.69 (d, *J* = 16.4 Hz, 1H), 2.97 (d, *J* = 16.5 Hz, 1H), 3.39 (d, *J* = 13.5 Hz, 1H), 3.51 (t, *J* = 8.6 Hz, 2H), 3.64 (d, *J* = 14.3 Hz, 1H), 4.85 (s, 1H), 6.89 (t, *J* = 8.6 Hz, 2H), 6.99 (t, *J* = 8.5 Hz, 2H), 7.04 – 7.10 (m, 3H), 7.10 – 7.13 (m, 2H), 7.16 (dd, *J* = 8.5, 5.3 Hz, 2H), 7.19 – 7.23 (m, 3H), 7.66 (brs, 1H), 8.77 (s, 1H), 9.90 (s, 1H).

^{13}C NMR (125 MHz, Chloroform-*d*) δ 51.61, 51.90, 55.86, 60.92, 110.62, 115.39 (d, J = 21.5 Hz), 116.37 (d, J = 21.7 Hz), 124.10, 125.53, 127.66, 128.47, 128.96 (d, J = 8.3 Hz), 129.13, 131.03 (d, J = 8.0 Hz), 131.79 (d, J = 2.8 Hz), 135.78 (d, J = 3.2 Hz), 136.69, 152.12, 162.04 (d, J = 248.2 Hz), 162.99 (d, J = 248.5 Hz). HPLC purity, 95.81. HRMS (m/z): $[M+H]^+$ calcd for $C_{27}H_{24}F_2N_4$, 443.2047; found, 443.2099.

5a

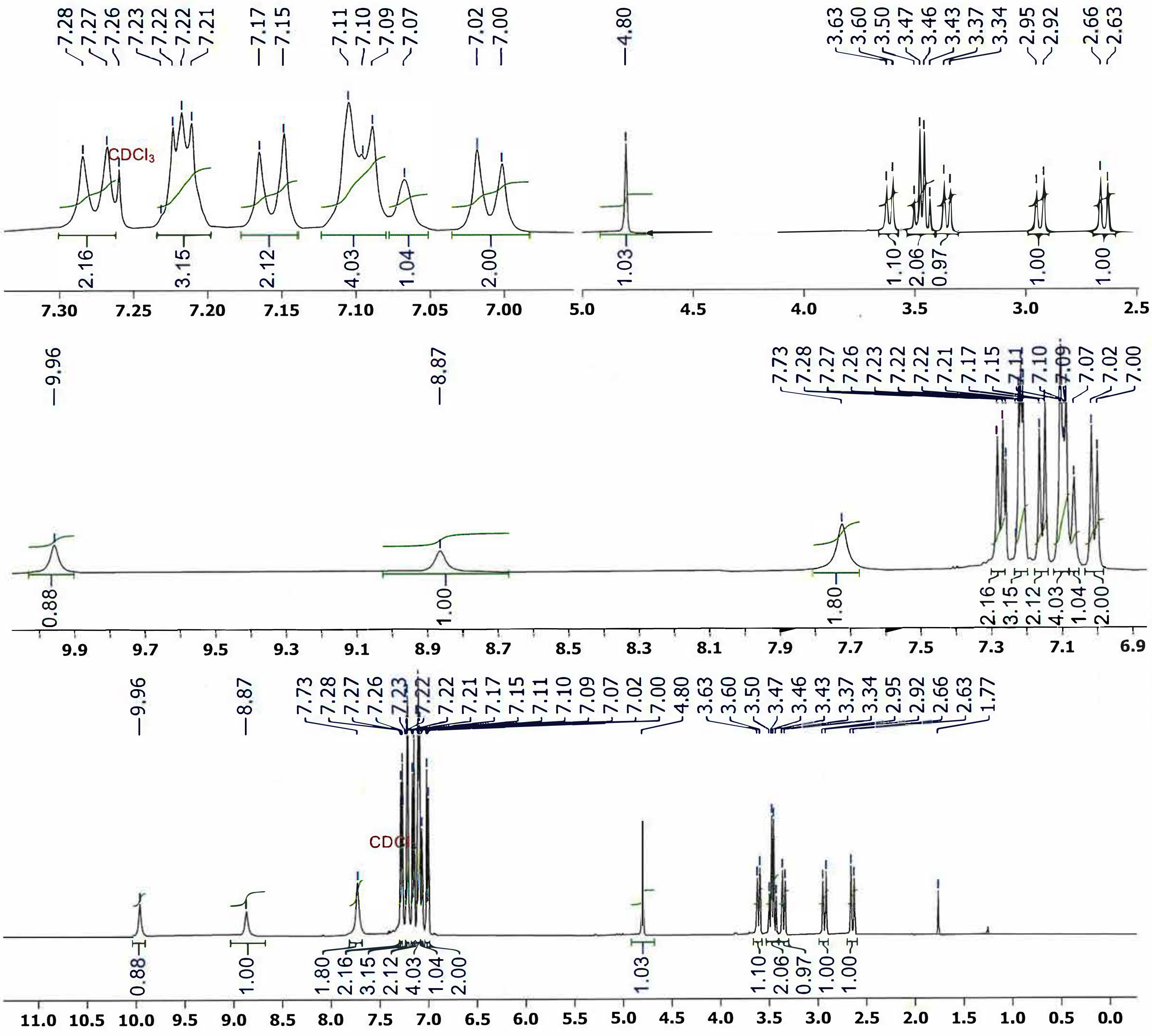

	Parámetro	Valor
1	Comment	No comment
2	Origin	Bruker BioSpin GmbH
3	Owner	ICON
4	Instrument	spect
5	Solvent	CDCl3
6	Temperature	298.0
7	Pulse Sequence	zg30
8	Experiment	1D
9	Probe	5 mm PABBO BB/ 19F-1H/ D Z-GRD Z113652/ 0203
10	Number of Scans	16
11	Receiver Gain	69.7
12	Relaxation Delay	6.0000
13	Pulse Width	10.0000
14	Acquisition Time	3.2855
15	Acquisition Date	2020-07-09T19:08:35
16	Modification Date	2020-07-09T19:10:00
17	Spectrometer Frequency	499.25
18	Spectral Width	9973.4
19	Lowest Frequency	-1915.7
20	Nucleus	1H
21	Acquired Size	32768
22	Spectral Size	65536
23	Digital Resolution	0.15

^{1}H NMR (499 MHz, Chloroform-*d*) δ = 2.65 (d, *J*=16.4, 1H, CH_2), 2.93 (d, *J*=16.5, 1H, CH_2), 3.35 (d, *J*=14.0, 1H, CH_2), 3.41 – 3.53 (m, 2H, CH_2Bn), 3.61 (d, *J*=14.0, 1H, CH_2), 4.80 (s, 1H, CH), 7.01 (d, *J*=8.2, 2H, H-Ar), 7.07 (s, 1H, CH=), 7.08 – 7.12 (m, 4H, H-Ar), 7.16 (d, *J*=8.1, 2H, H-Ar), 7.20 – 7.23 (m, 3H, H-Ar), 7.28 (d, *J*=8.1, 2H, H-Ar), 7.73 (s, 1H, NH), 8.87 (s, 1H, NH), 9.96 (s, 1H, NH).

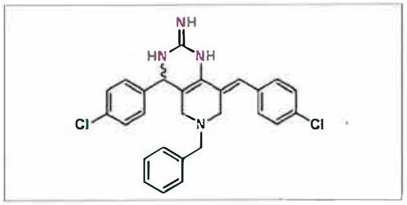

Title: AUD1-89

Molecular Formula

$C_{27}H_{24}Cl_2N_4$

Author:

Dr. Audifás-Salvador Matus-Meza

5a

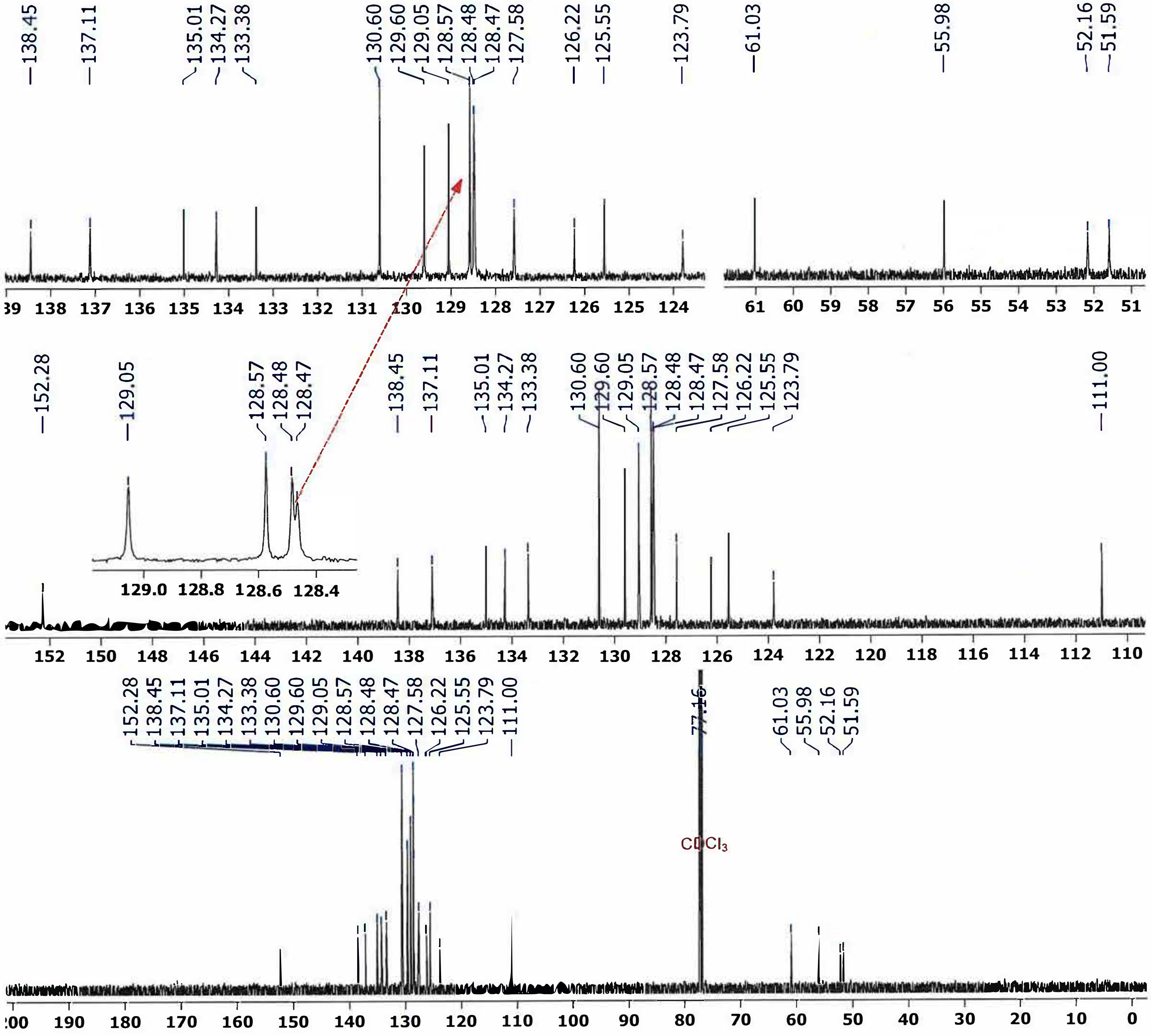

	Parámetro	Valor
1	Comment	No comment
2	Origin	Bruker BioSpin GmbH
3	Owner	ICON
4	Instrument	spect
5	Solvent	CDCl3
6	Temperature	298.0
7	Pulse Sequence	zgpg30
8	Experiment	1D
9	Probe	5 mm PABBO BB/ 19F-1H/ D Z-GRD Z113652/ 0203
10	Number of Scans	240
11	Receiver Gain	191.5
12	Relaxation Delay	1.0000
13	Pulse Width	10.0000
14	Acquisition Time	1.1010
15	Acquisition Date	2020-07-10T13:15:31
16	Modification Date	2020-07-10T13:22:00
17	Spectrometer Frequency	125.55
18	Spectral Width	29761.9
19	Lowest Frequency	-2315.9
20	Nucleus	13C
21	Acquired Size	32768
22	Spectral Size	65536
23	Digital Resolution	0.45

^{13}C NMR (126 MHz, Chloroform-*d*) δ = 51.59, 52.16, 55.98, 61.03, 111.00, 123.79, 125.55, 126.22, 127.58, 128.47, 128.48, 128.57, 129.05, 129.60, 130.60, 133.38, 134.27, 135.01, 137.11, 138.45, 152.28.

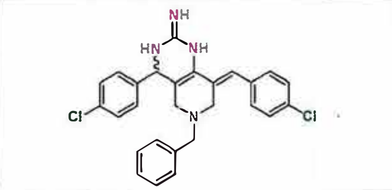

Title: AUD1-89
Molecular Formula
$C_{27}H_{24}Cl_2N_4$
Author:
Dr. Audifás-Salvador Matus-Meza

5a

===

Acq. Operator : SYSTEM Seq. Line : 4
Acq. Instrument : 1220 HPLC Location : 22
Injection Date : 9/9/2020 9:55:22 AM Inj : 1
Inj Volume : 30.000 µl
Different Inj Volume from Sample Entry! Actual Inj Volume : 50.000 µl
Method : C:\Chem32\1\Data\Audifas 2020-09-09 08-32-40\MOHAMED-SMALL MOLECULE.M (Sequence Method)
Last changed : 9/9/2020 8:32:43 AM by SYSTEM
Method Info : Water/MeOH

Sample Info : AUD1-89

Additional Info : Peak(s) manually integrated

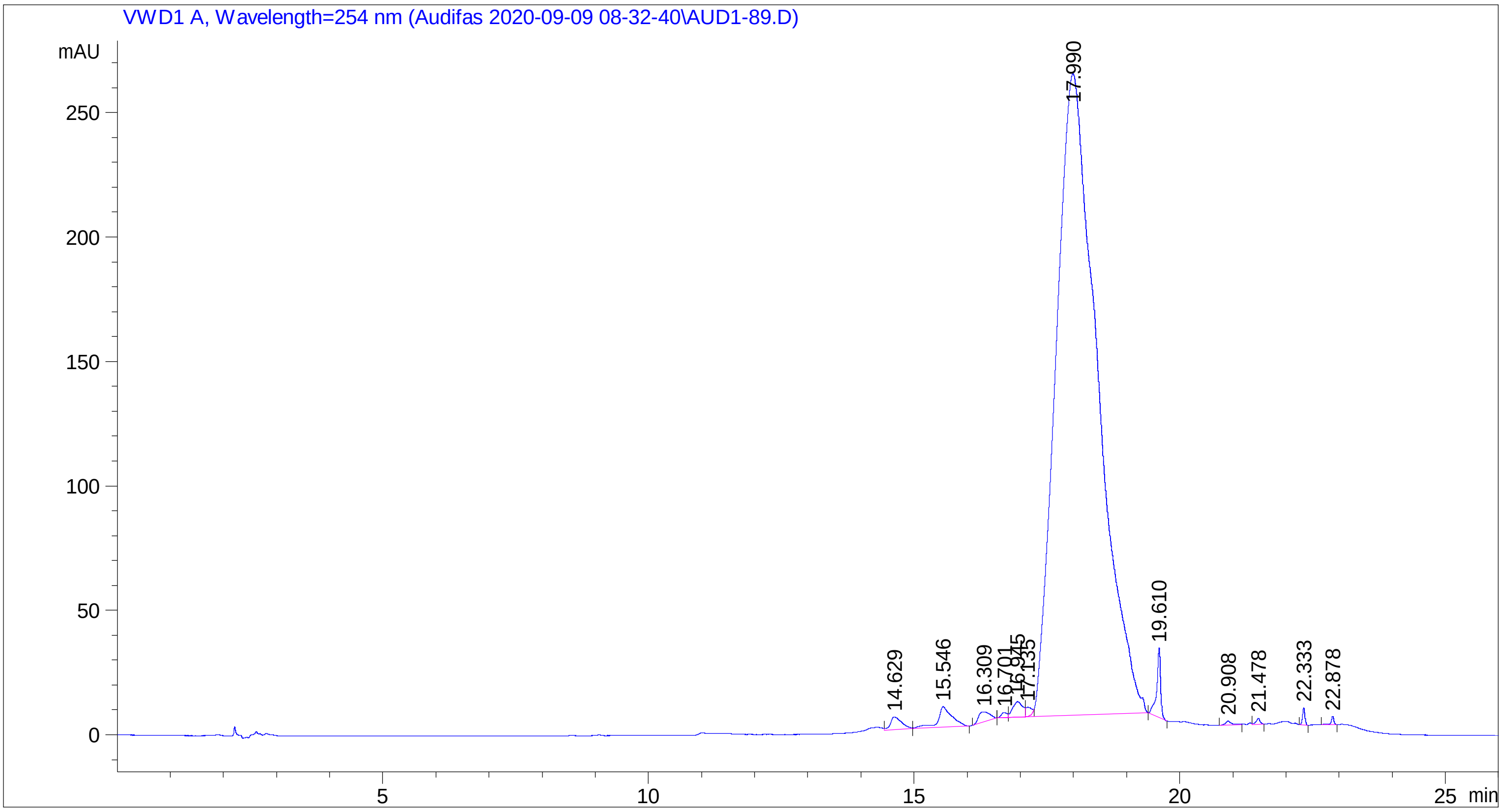

===

Area Percent Report

===

Sorted By : Signal
Multiplier : 1.0000
Dilution : 1.0000
Use Multiplier & Dilution Factor with ISTDs

Signal 1: VWD1 A, Wavelength=254 nm

Peak #	RetTime [min]	Type	Width [min]	Area [mAU*s]	Height [mAU]	Area %
1	14.629	VB	0.1712	72.17616	5.09686	0.4959
2	15.546	VB R	0.2301	148.65898	8.15733	1.0213
3	16.309	BB	0.1730	59.13889	4.04560	0.4063
4	16.701	BV E	0.0957	16.13327	2.09819	0.1108

S9

Peak #	RetTime [min]	Type	Width [min]	Area [mAU*s]	Height [mAU]	Area %
5	16.945	VV E	0.1590	82.35879	6.27986	0.5658
6	17.135	VV E	0.0870	27.26900	3.69880	0.1873
7	17.990	VV R	0.6967	1.39429e4	257.90570	95.7885
8	19.610	BB	0.0745	144.94482	27.94933	0.9958
9	20.908	BV R	0.0935	13.58034	1.80022	0.0933
10	21.478	VB	0.0712	13.27092	2.53137	0.0912
11	22.333	BB	0.0490	22.44610	7.07464	0.1542
12	22.878	VB R	0.0554	13.04629	3.46551	0.0896

Totals : 1.45559e4 330.10341

===

*** End of Report ***

S10

5a

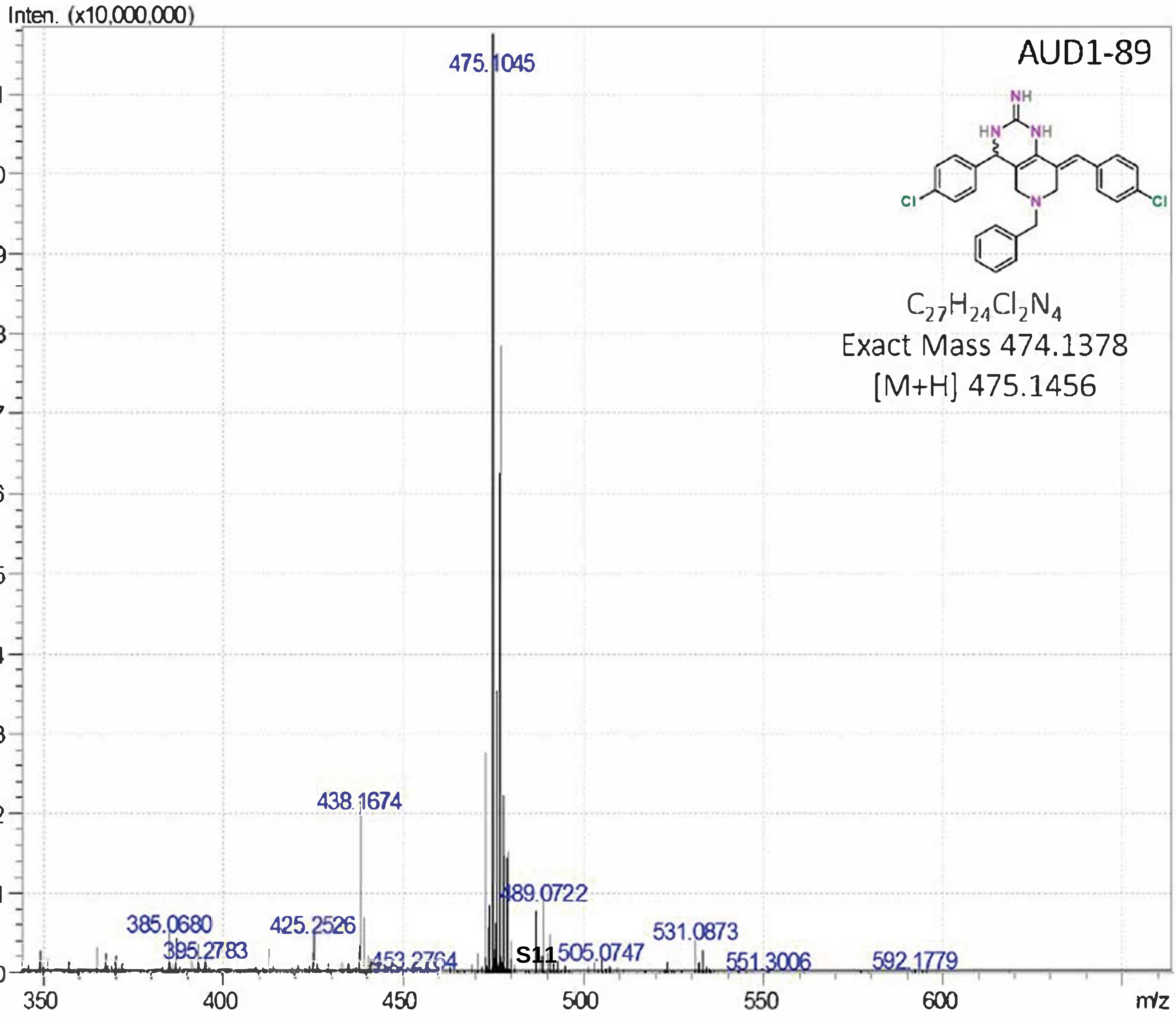

Inten. (x10,000,000)
AUD1-89
NH
HN
NH
Cl
Cl
N
$C_{27}H_{24}Cl_2N_4$
Exact Mass 474.1378
[M+H] 475.1456
475.1045
438.1674
489.0722
385.0680
425.2526
395.2783
453.2764
505.0747
531.0873
551.3006
592.1779
1.1
1.0
0.9
0.8
0.7
0.6
0.5
0.4
0.3
0.2
0.1
0.0
350
400
450
500
550
600
m/z

S11

5b

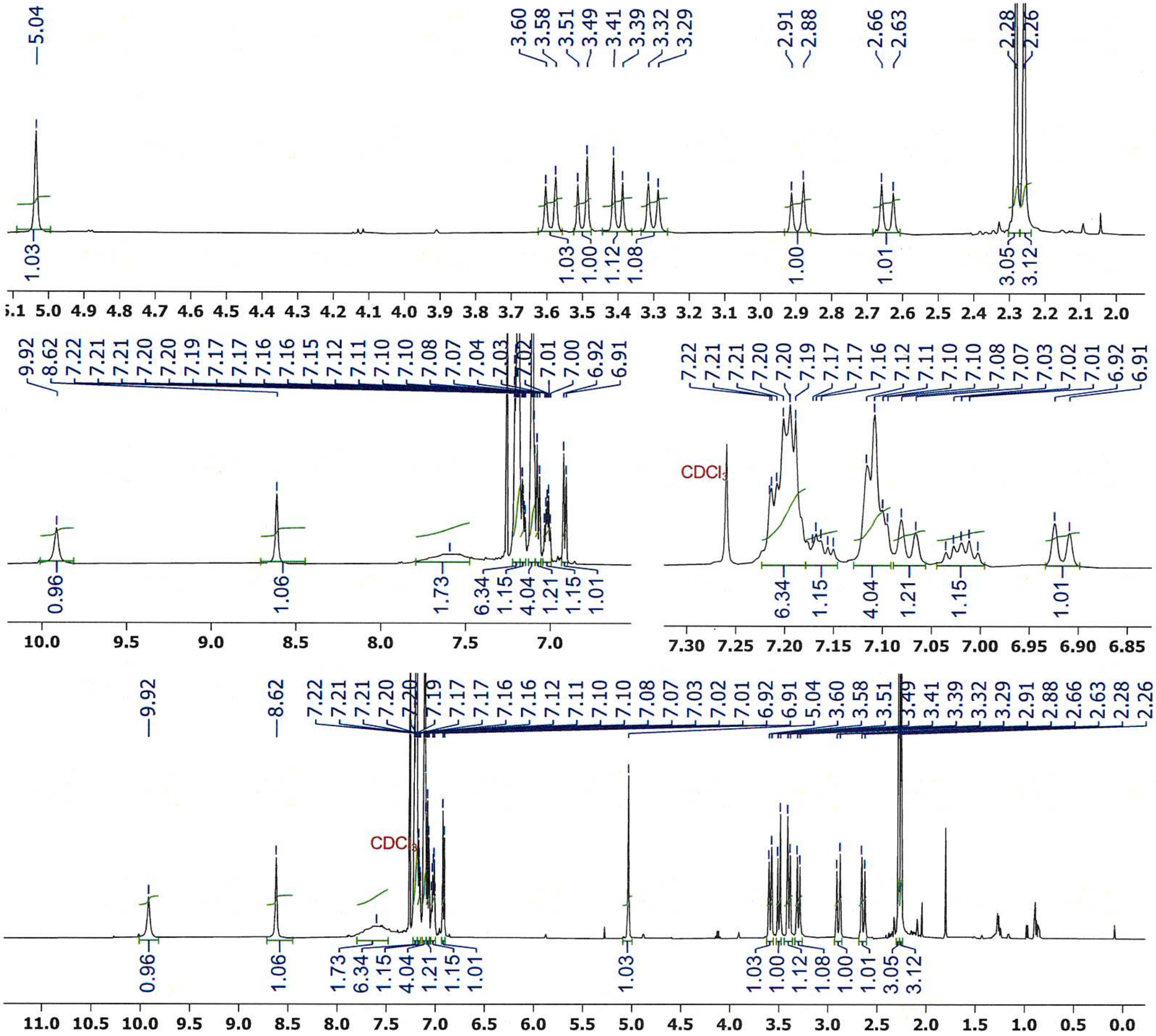

Parámetro		Valor
1	Comment	No comment
2	Origin	Bruker BioSpin GmbH
3	Owner	ICON
4	Instrument	spect
5	Solvent	CDCl3
6	Temperature	298.0
7	Pulse Sequence	zg30
8	Experiment	1D
9	Probe	5 mm PABBO BB/ 19F-1H/ D Z-GRD Z113652/ 0203
10	Number of Scans	16
11	Receiver Gain	29.8
12	Relaxation Delay	1.0000
13	Pulse Width	10.0000
14	Acquisition Time	3.2855
15	Acquisition Date	2020-07-15T15:18:36
16	Modification Date	2020-07-15T15:18:00
17	Spectrometer Frequency	499.25
18	Spectral Width	9973.4
19	Lowest Frequency	-1915.7
20	Nucleus	1H
21	Acquired Size	32768
22	Spectral Size	65536
23	Digital Resolution	0.15

^{1}H NMR (499 MHz, Chloroform-*d*) δ = 2.26 (s, 3H), 2.28 (s, 3H), 2.64 (d, *J*=16.4, 1H), 2.90 (d, *J*=16.6, 1H), 3.30 (d, *J*=13.9, 1H), 3.40 (d, *J*=12.9, 1H), 3.50 (d, *J*=12.8, 1H), 3.59 (d, *J*=13.9, 1H), 5.04 (s, 1H), 6.92 (d, *J*=7.6, 1H), 7.02 (dt, *J*=8.6, 4.2, 1H, H-Ar), 7.07 (d, *J*=7.4, 1H, H-Ar), 7.11 (m, 4H, H-Ar), 7.16 (m, 1H, H-Ar), 7.20 (m, 6H, H-Ar), 7.59 (s, 1H, NH), 8.62 (s, 1H, NH), 9.92 (s, 1H, NH).

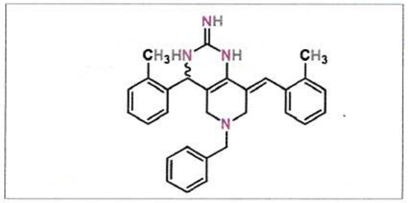

Title: **AUD1-90**

Molecular Formula

$C_{29}H_{30}N_4$

Author:

Dr. Audifás-Salvador Matus-Meza

5b

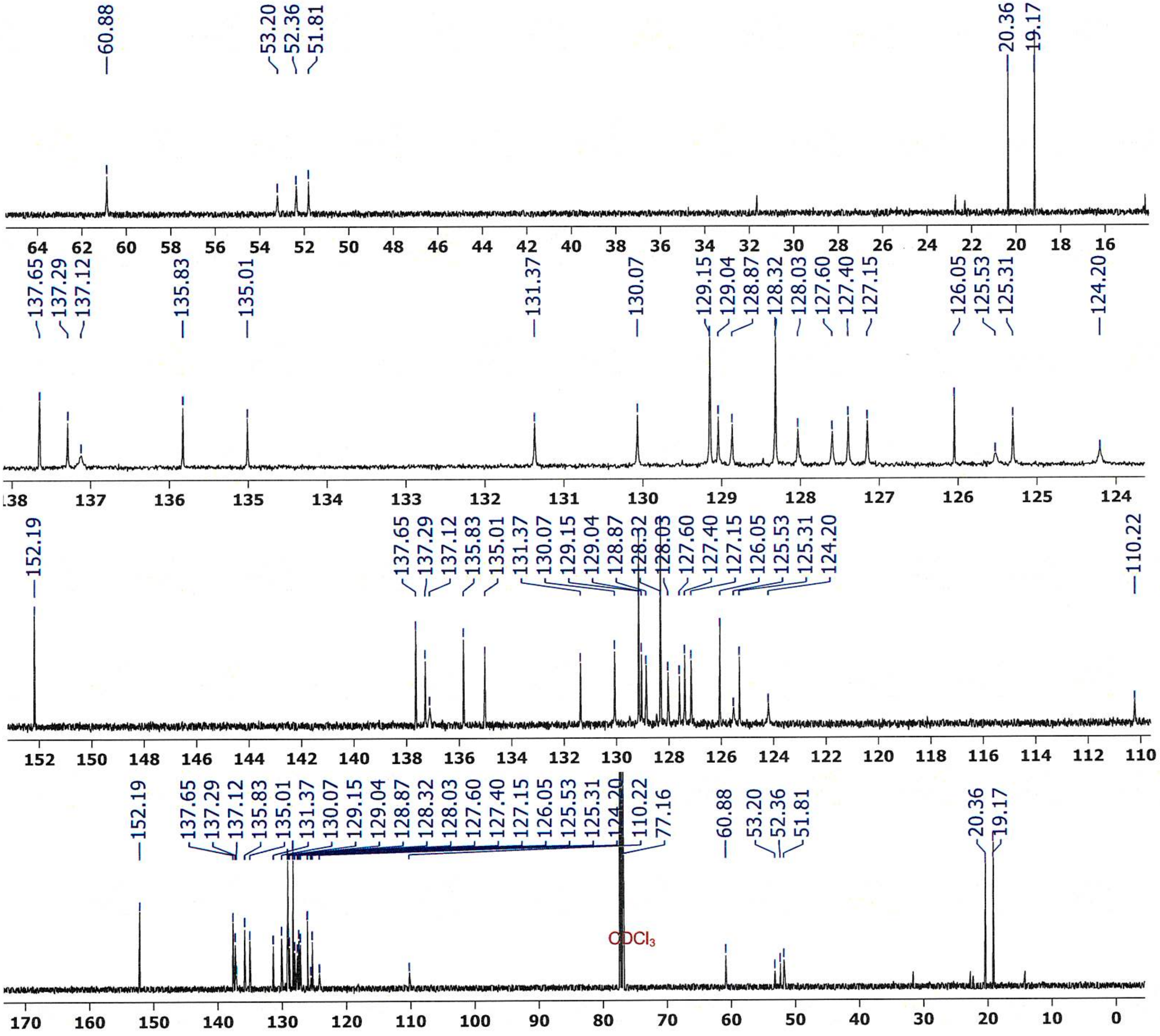

Parámetro		Valor
1	Comment	No comment
2	Origin	Bruker BioSpin GmbH
3	Owner	ICON
4	Instrument	spect
5	Solvent	CDCl3
6	Temperature	298.0
7	Pulse Sequence	zgpg30
8	Experiment	1D
9	Probe	5 mm PABBO BB/ 19F-1H/ D Z-GRD Z113652/ 0203
10	Number of Scans	294
11	Receiver Gain	191.5
12	Relaxation Delay	1.0000
13	Pulse Width	10.0000
14	Acquisition Time	1.1010
15	Acquisition Date	2020-07-15T15:22:25
16	Modification Date	2020-07-15T15:30:00
17	Spectrometer Frequency	125.55
18	Spectral Width	29761.9
19	Lowest Frequency	-2322.5
20	Nucleus	13C
21	Acquired Size	32768
22	Spectral Size	65536
23	Digital Resolution	0.45

S13

^{13}C NMR (126 MHz, Chloroform-*d*) δ = 19.17, 20.36, 51.81, 52.36, 53.20, 60.88, 110.22, 124.20, 125.31, 125.53, 126.05, 127.15, 127.40, 127.60, 128.03, 128.32, 128.87, 129.04, 129.15, 130.07, 131.37, 135.01, 135.83, 137.12, 137.29, 137.65,

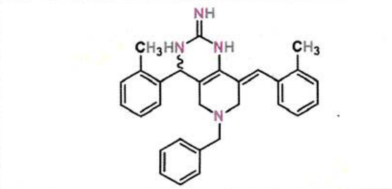

Title: **AUD1-90**

Molecular Formula

$C_{29}H_{30}N_4$

Author:

Dr. Audifás-Salvador Matus-Meza

5b

===

Acq. Operator : SYSTEM Seq. Line : 3
Acq. Instrument : 1220 HPLC Location : 31
Injection Date : 9/7/2020 2:26:13 PM Inj : 1
Inj Volume : 30.000 µl
Different Inj Volume from Sample Entry! Actual Inj Volume : 50.000 µl
Method : C:\Chem32\1\Data\Audifas 2020-09-07 13-31-15\MOHAMED-SMALL MOLECULE.M (Sequence Method)
Last changed : 9/7/2020 2:12:59 PM by SYSTEM
Method Info : Water/MeOH

Sample Info : AUD1-90

Additional Info : Peak(s) manually integrated

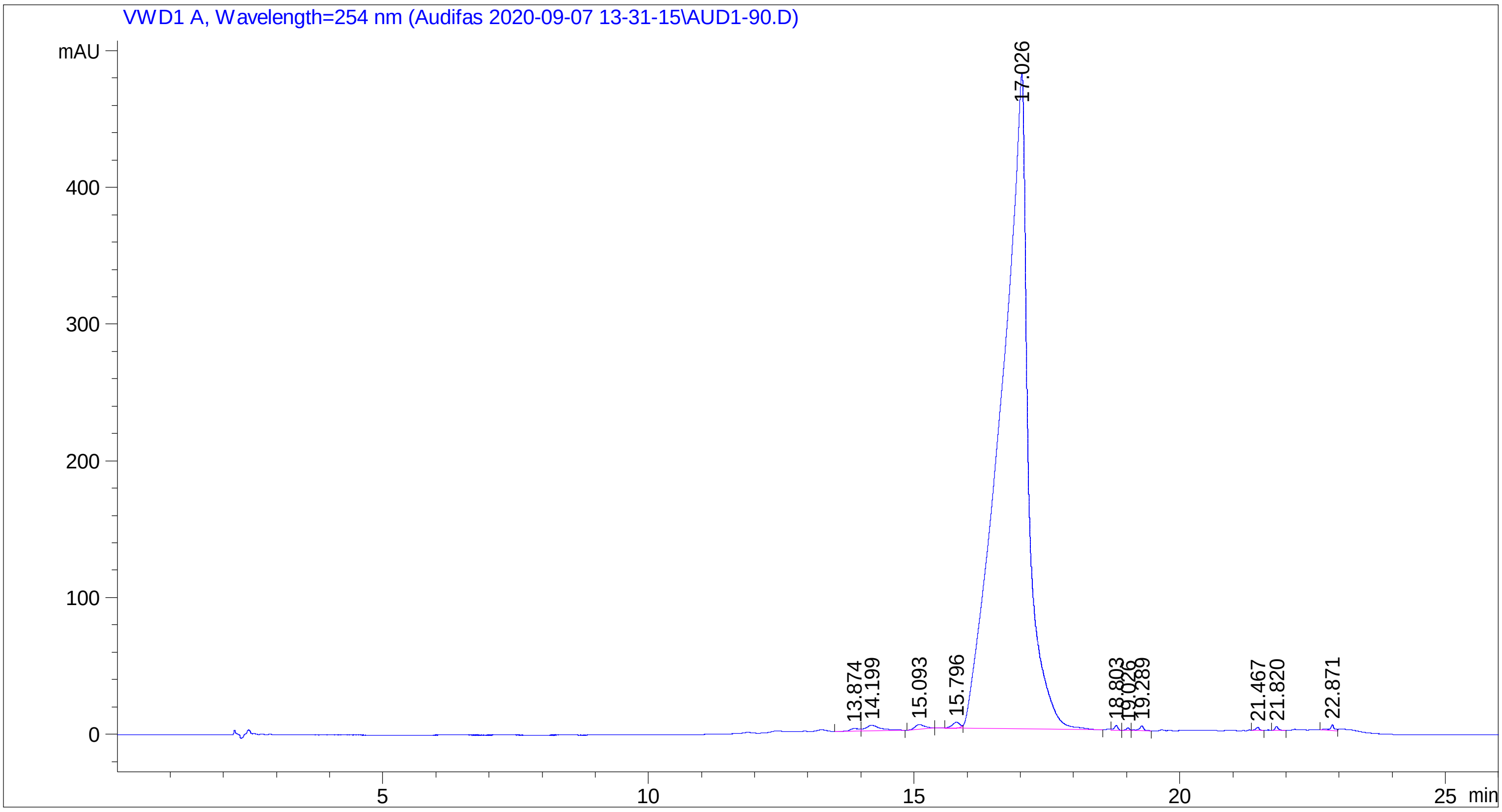

===

Area Percent Report

===

Sorted By : Signal
Multiplier : 1.0000
Dilution : 1.0000
Use Multiplier & Dilution Factor with ISTDs

Signal 1: VWD1 A, Wavelength=254 nm

Peak #	RetTime [min]	Type	Width [min]	Area [mAU*s]	Height [mAU]	Area %
1	13.874	BV	0.1420	23.95087	2.00242	0.1373
2	14.199	VV R	0.2358	83.81377	4.20871	0.4806
3	15.093	BB	0.1552	45.47578	3.46391	0.2608
4	15.796	VV E	0.1165	42.97590	4.36166	0.2464

Peak #	RetTime [min]	Type	Width [min]	Area [mAU*s]	Height [mAU]	Area %
5	17.026	VV R	0.4537	1.71483e4	479.49423	98.3374
6	18.803	VB	0.0675	16.75790	3.58563	0.0961
7	19.026	BV	0.0610	7.88982	1.82445	0.0452
8	19.289	VB R	0.0807	20.94196	3.66558	0.1201
9	21.467	VB	0.0682	11.74576	2.33267	0.0674
10	21.820	BV R	0.0583	11.70019	2.97726	0.0671
11	22.871	VV R	0.0773	24.67612	4.30872	0.1415

Totals :				1.74382e4	512.22524	

*** End of Report ***

5b

Spectrum Plot Report

Name	Aud1-90	Rack Pos.		Instrument	Instrument 1	Operator	
Inj. Vol. (ul)	1	Plate Pos.		IRM Status	Some ions missed		
Data File	Aud1-90-1.d	Method (Acq)	Full gradient organic.m	Comment	5-95% ACN in 3 min; hold 2 min	Acq. Time (Local)	2/19/2021 3:57:48 PM (UTC-06:00)

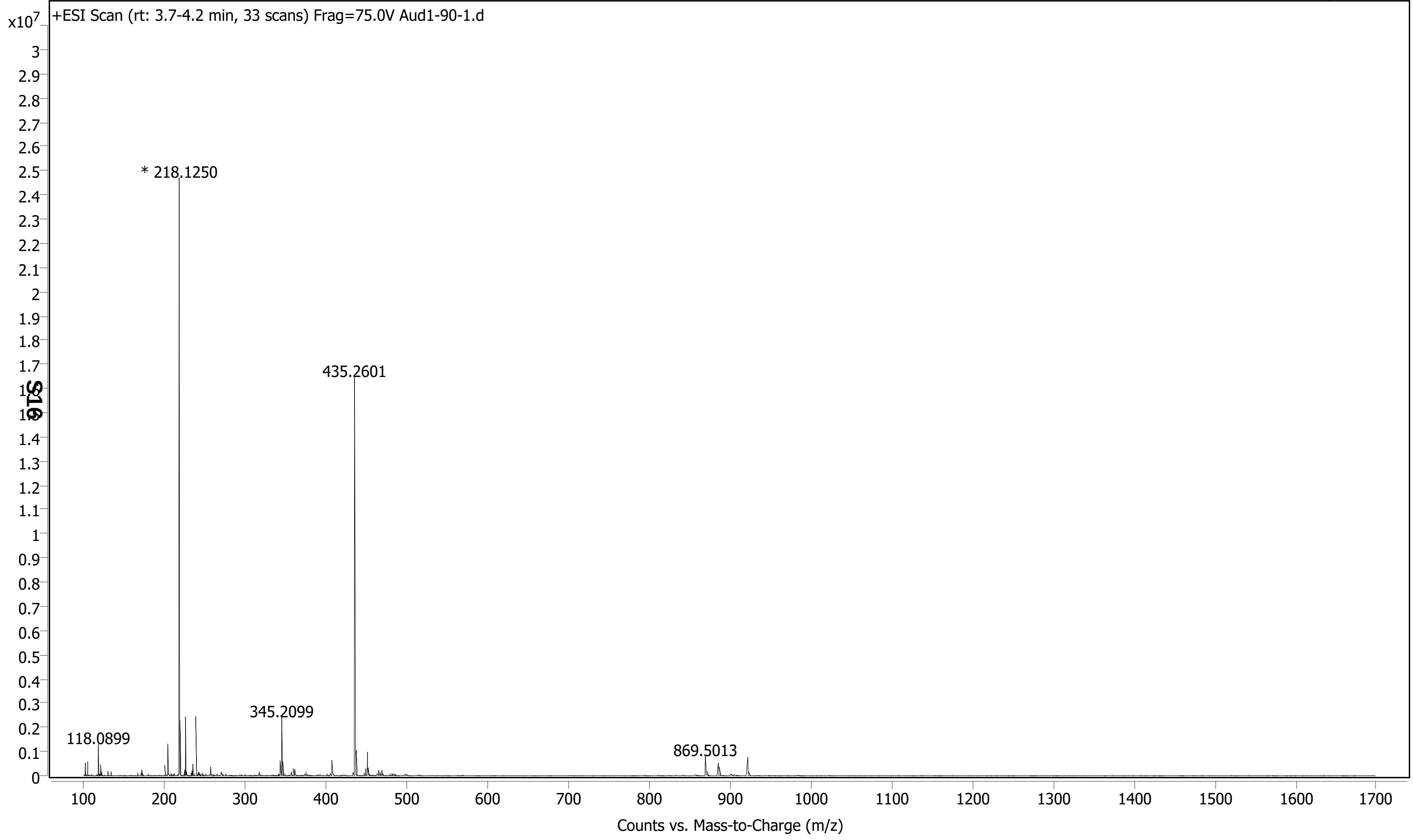

S16

Generated at 6:18 PM on 2/19/2021

5c

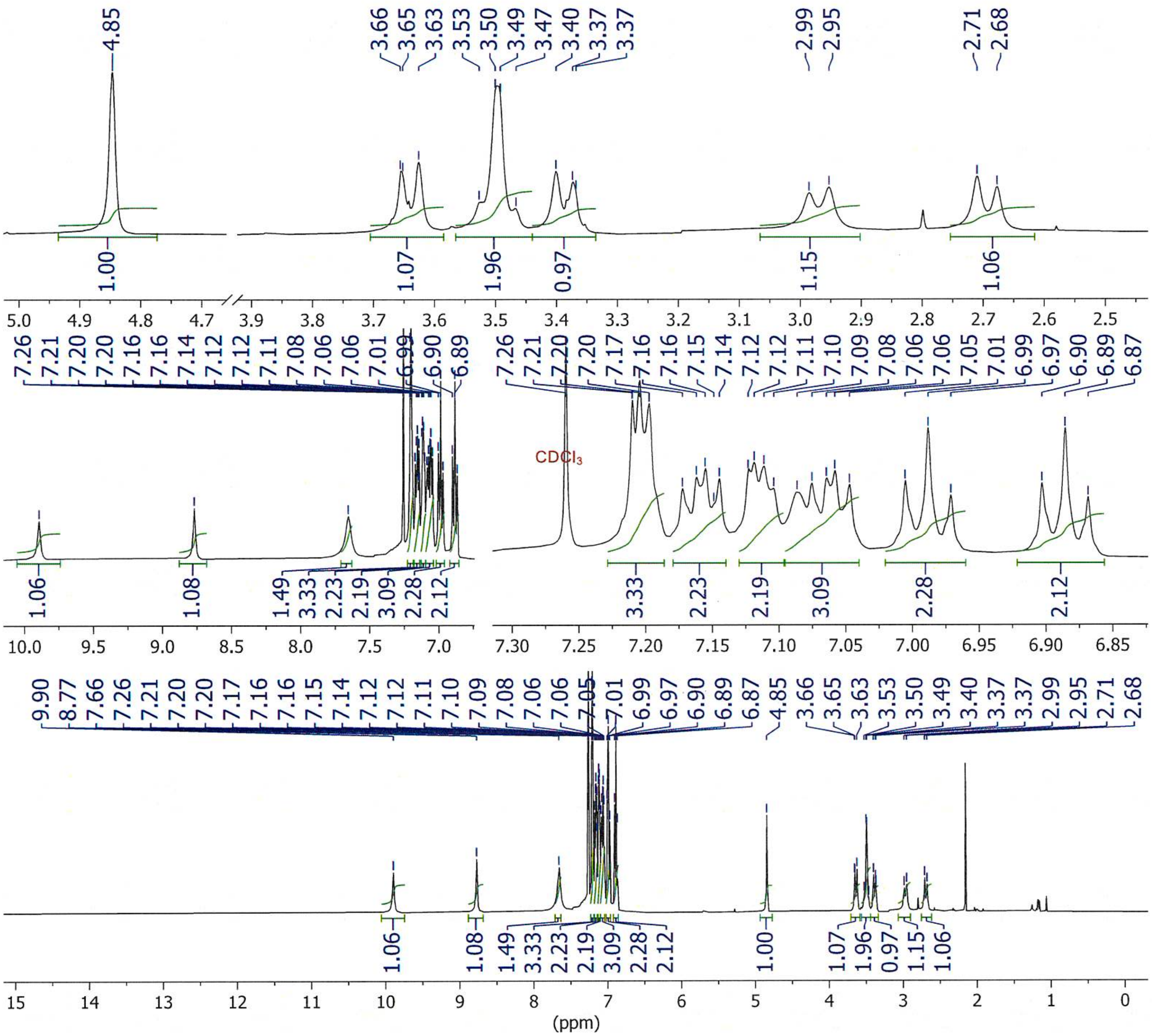

	Parameter	Value
1	Comment	Name registered in the lab notebook: **AUD3-60**
2	Origin	Bruker BioSpin GmbH
3	Owner	ICON
4	Solvent	CDCl3
5	Temperature	298.0
6	Pulse Sequence	zg30
7	Experiment	1D
8	Probe	5 mm PABBO BB/ 19F-1H/ D Z-GRD Z113652/ 0203
9	Number of Scans	16
10	Receiver Gain	49.0
11	Relaxation Delay	6.0000
12	Pulse Width	10.0000
13	Acquisition Time	3.2855
14	Acquisition Date	2020-12-11T19:01:00
15	Modification Date	2020-12-11T19:01:00
16	Spectrometer Frequency	499.23
17	Spectral Width	9973.4
18	Lowest Frequency	-1915.5
19	Nucleus	1H
20	Acquired Size	32768
21	Spectral Size	65536
22	Digital Resolution	0.15

^{1}H NMR (499 MHz, Chloroform-*d*) δ 2.6[illegible] (d, *J* = 16.4 Hz, 1H), 2.97 (d, *J* = 16.5 Hz, 1H), 3.39 (d, *J* = 13.5 Hz, 1H), 3.51 (t, *J* = 8.6 Hz, 2H), 3.64 (d, *J* = 14.3 Hz, 1H), 4.85 (s, 1H), 6.89 (t, *J* = 8.6 Hz, 2H), 6.99 (t, *J* = 8.5 Hz, 2H), 7.04 – 7.10 (m, 3H), 7.10 – 7.13 (m, 2H), 7.16 (dd, *J* = 8.5, 5.3 Hz, 2H), 7.19 – 7.23 (m, 3H), 7.66 (brs, 1H), 8.77 (s, 1H), 9.90 (s, 1H).

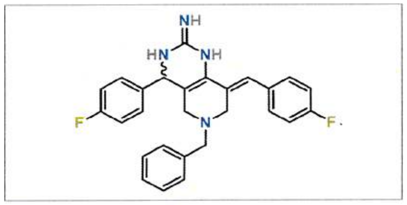

Title: **AUD3-60**

Molecular Formula

$C_{27}H_{24}F_2N_4$

Author:

Dr. Audifás-Salvador Matus-Meza

5c

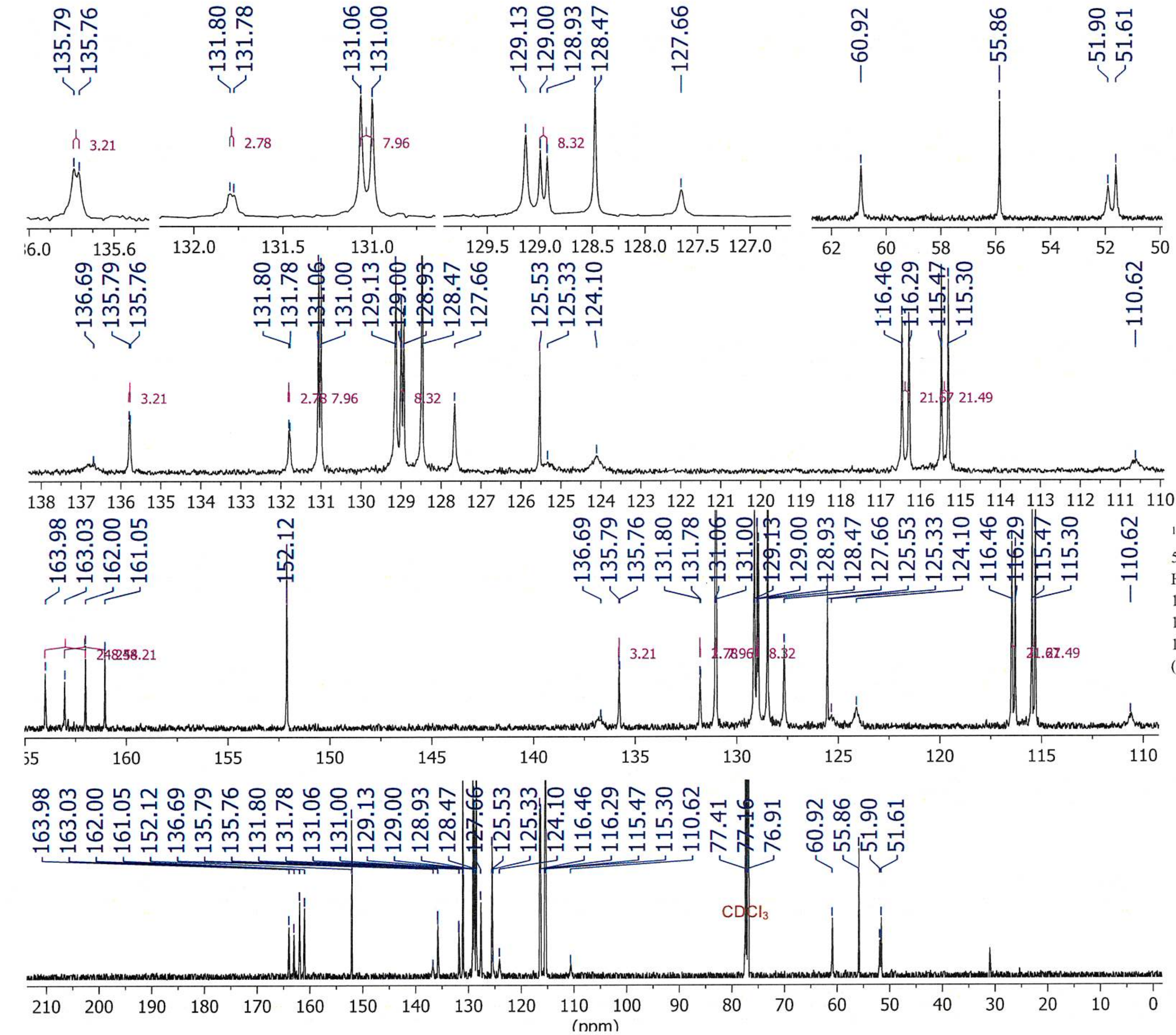

	Parameter	Value
1	Comment	Name registered in the lab notebook: **AUD3-60**
2	Origin	Bruker BioSpin GmbH
3	Owner	ICON
4	Solvent	CDCl3
5	Temperature	298.0
6	Pulse Sequence	zgpg30
7	Experiment	1D
8	Probe	5 mm PABBO BB/ 19F-1H/ D Z-GRD Z113652/ 0203
9	Number of Scans	1024
10	Receiver Gain	191.5
11	Relaxation Delay	1.0000
12	Pulse Width	10.0000
13	Acquisition Time	1.1010
14	Acquisition Date	2020-12-11T19:39:13
15	Modification Date	2020-12-11T19:39:00
16	Spectrometer Frequency	125.54
17	Spectral Width	29761.9
18	Lowest Frequency	-2317.7
19	Nucleus	13C
20	Acquired Size	32768
21	Spectral Size	65536
22	Digital Resolution	0.45

^{13}C NMR (126 MHz, Chloroform-*d*) δ 51.61, 51.90, 55.86, 60.92, 110.62, 115.39 (d, *J* = 21.5 Hz), 116.37 (d, *J* = 21.7 Hz), 124.10, 125.53, 127.66, 128.47, 128.96 (d, *J* = 8.3 Hz), 129.13, 131.03 (d, *J* = 8.0 Hz), 131.79 (d, *J* = 2.8 Hz), 135.78 (d, *J* = 3.2 Hz), 136.69, 152.12, 162.04 (d, *J* = 248.2 Hz), 162.99 (d, *J* = 248.5 Hz).

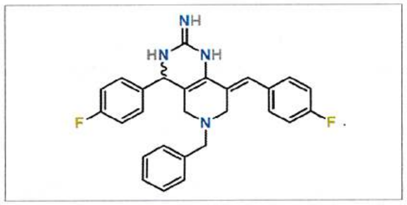

Title: **AUD3-60**

Molecular Formula

$C_{27}H_{24}F_2N_4$

Author:

Dr. Audifás-Salvador Matus-Meza

Data File C:\Chem32\1\Data\Mohamed 2020-12-14 10-16-59\AUD3-60-again.D
Sample Name: AUD3-60-again

5c

===

Acq. Operator : SYSTEM	Seq. Line : 9
Acq. Instrument : 1220 HPLC	Location : 62
Injection Date : 12/14/2020 2:04:54 PM	Inj : 1
	Inj Volume : 50.000 µl

Method : C:\Chem32\1\Data\Mohamed 2020-12-14 10-16-59\Audi-Method-Extend.M (Sequence Method)
Last changed : 12/14/2020 1:05:02 PM by SYSTEM
Method Info : Water/Methanol

Sample Info : AUD3-60-again

Additional Info : Peak(s) manually integrated

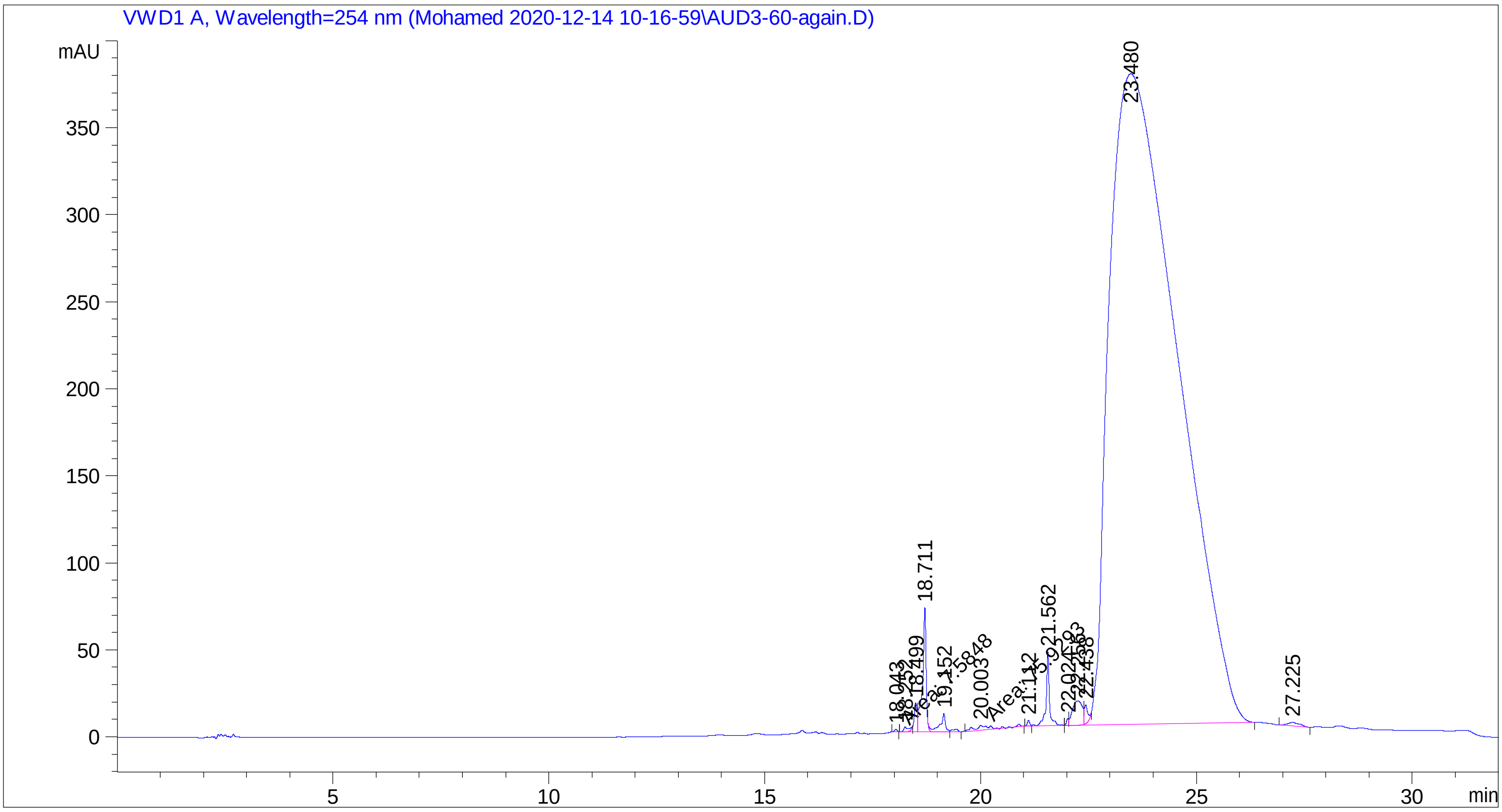

===
Area Percent Report
===

Sorted By : Signal
Multiplier : 1.0000
Dilution : 1.0000
Use Multiplier & Dilution Factor with ISTDs

Signal 1: VWD1 A, Wavelength=254 nm

Peak #	RetTime [min]	Type	Width [min]	Area [mAU*s]	Height [mAU]	Area %
1	18.043	MM T	0.0777	7.58480	1.62664	0.0181
2	18.252	BV E	0.1032	21.66106	2.77037	0.0517
3	18.499	VV R	0.0735	85.24936	16.70203	0.2034
4	18.711	VV R	0.0849	432.93301	71.28183	1.0328
5	19.152	VV E	0.1186	93.31518	10.40668	0.2226

S19

Peak #	RetTime [min]	Type	Width [min]	Area [mAU*s]	Height [mAU]	Area %
6	20.003	MM T	0.4272	75.92931	2.96207	0.1811
7	21.112	BV E	0.0619	13.91825	3.39194	0.0332
8	21.562	VV R	0.0775	232.18285	42.68695	0.5539
9	22.024	BV E	0.0578	15.02681	4.00148	0.0358
10	22.256	VV E	0.2584	220.48619	13.94657	0.5260
11	22.438	VV E	0.0771	59.63864	10.85810	0.1423
12	23.480	VB R	1.5498	4.06185e4	373.77307	96.8990
13	27.225	BB	0.2709	41.96278	2.11143	0.1001
Totals :				4.19184e4	556.51916	

==

*** End of Report ***

5c

Spectrum Plot Report

Name	Aud3-60	Rack Pos.		Instrument	Instrument 1	Operator	
Inj. Vol. (ul)	1	Plate Pos.		IRM Status	Some ions missed		
Data File	Aud3-60-2.d	Method (Acq)	Full gradient organic.m	Comment	5-95% ACN in 3 min; hold 2 min pos	Acq. Time (Local)	2/19/2021 7:26:36 PM (UTC-06:00)

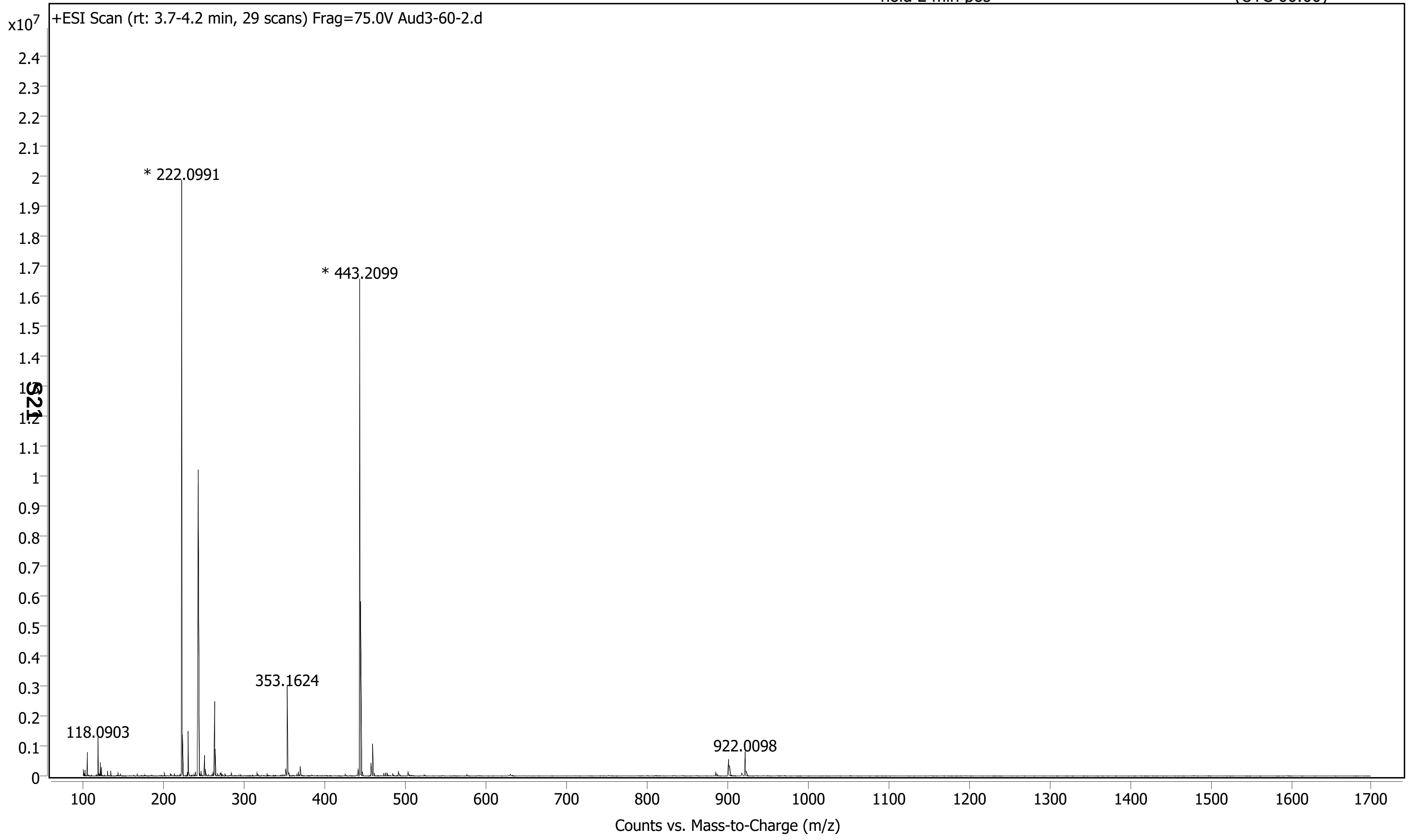

S21

Generated at 7:46 PM on 2/19/2021

Table S1. Minimum Inhibitory Concentration (μg.mL^{-1}) of compounds against Gram-positive and Gram-negative strains

		**5a**	**5b**	**5c**	Antibiotic
**Gram-positive**	*Bacillus cereus* 11778	1.17	4.68	4.68	0.25[a]
	Staphylococcus saprophyticus BAA750	37.5	9.37	37.5	>128[a]
	Staphylococcus aureus 29213	2.34	2.34	2.34	0.06[a]
	CA-MRSA	4.68	2.34	4.68	16[b]
	HA-MRSA	4.68	2.34	4.68	32[b]
**Gram-negative**	*Salmonella* Typhimurium 14028	75	150	37.5	1[a]
	Klebsiella pneumoniae 700603	37.5	9.37	37.5	>128[a]

[a]: ampicillin [b]: oxacillin

Table S2. Minimum inhibitory concentration (μg/mL) of compounds after treatment with different conditions

		Cont	NaCl	$CaCl_2$	KCl	FBS 2%	FBS 5%	FBS 10%	HP 2%	HP 5%	HP 50%	HS 2%	HS 5%	HS 50%	pH 4	pH 5	pH 8	BSA 2%	BSA 5%	BSA 10%	Trip
**5a**	*S. aureus*	2.34	2.34	18.72	37.44	9.36	9.36	9.36	9.36	18.72	18.72[a]<br>37.44[b]	9.36	18.72	18.72[a]<br>37.44[b]	9.36	9.36	9.36	2.34	2.34	2.34	2.34
	CA-MRSA	4.68	4.68	18.72	37.44	9.36	9.36	9.36	9.36	18.72	18.72[a]<br>37.44[b]	18.72	18.72	18.72[a]<br>37.44[b]	9.36	9.36	9.36	4.68	4.68	4.68	4.68
	HA-MRSA	4.68	4.68	18.72	37.44	9.36	9.36	9.36	9.36	18.72	18.72[a]<br>18.72[b]	18.72	18.72	18.72[a]<br>37.44[b]	9.36	9.36	9.36	4.68	4.68	4.68	4.68
**5b**	*S. aureus*	2.34	2.34	9.36	9.36	4.68	4.68	4.68	4.68	9.36	9.36[a]<br>18.72[b]	9.36	9.36	9.36[a]<br>18.72[b]	9.36	9.36	9.36	4.68	4.68	4.68	2.34
	CA-MRSA	2.34	2.34	9.36	9.36	4.68	4.68	4.68	9.36	9.36	9.36[a]<br>9.36[b]	9.36	9.36	9.36[a]<br>18.72[b]	9.36	9.36	9.36	2.34	2.34	2.34	2.34
	HA-MRSA	2.34	2.34	9.36	9.36	4.68	4.68	4.68	9.36	9.36	9.36[a]<br>9.36[b]	9.36	9.36	9.36[a]<br>18.72[b]	9.36	9.36	18.72	2.34	2.34	2.34	2.34
**5c**	*S. aureus*	2.34	9.36	9.36	4.68	4.68	4.68	9.36	4.68	9.36	18.72[a]<br>18.72[b]	4.68	4.68	9.36[a]<br>37.44[b]	4.68	9.36	9.36	9.36	9.36	9.36	4.68
	CA-MRSA	4.68	4.68	4.68	4.68	4.68	4.68	9.36	4.68	9.36	18.72[a]<br>18.72[b]	4.68	4.68	9.36[a]<br>37.44[b]	9.36	9.36	18.72	9.36	9.36	4.68	9.36
	HA-MRSA	4.68	9.36	4.68	4.68	4.68	4.68	9.36	4.68	9.36	18.72[a]<br>18.72[b]	4.68	4.68	9.36[a]<br>37.44[b]	9.36	9.36	18.72	9.36	4.68	4.68	4.68

	S. aureus	2	2	2	4	2	2	4	2	4	4[a] 8[b]	4	4	4[a] 8[b]	2	2	2	4	4	4	4
**Oxa**	CA-MRSA	16	16	16	16	16	16	32	32	32	32[a] 32[b]	32	32	32[a] 64[b]	32	16	16	32	32	32	32
	HA-MRSA	32	32	32	32	32	64	64	64	64	64[a] 64[b]	64	64	64[a] 128[b]	32	32	32	64	64	64	64

Cont: control; FBS: fetal bovine serum; HP: human plasm; HS: human serum; BSA: bovine serum albumin; TRIP: tripsin; [a]: 12 hours pré-incubation [b]: 24 hours pré-incubation.

Table S3. Mutagenic activity expressed by mean revertants/plate ± standard deviation of compounds against strains TA98, TA100, TA102 of *S.* Typhimurium with metabolic activation (S+) and without metabolic activation (S-)

		TA98		TA100		TA102	
	μg/plate	S+	S-	S+	S-	S+	S-
**5a**	50	24.5 ± 4.9 (1.03)	23.0 ± 2.8 (1.02)	118.5 ± 6.3 (0.84)	147.5 ± 7.7 (0.93)	73.6 ± 2.8 (0.74)	73.6 ± 2.8 (0.74)
	150	26.0 ± 3.0 (1.10)	32.0 ± 3.6 (1.42)*	141.0 ± 16.9 (1.00)	132.0 ± 8.4 (0.84)	84.0 ± 1.0 (0.84)	84.0 ± 1.0 (0.84)
	500	26.5 ± 0.7 (1.12)*	32.0 ± 1.0 (1.42)	162.5 ± 4.9 (1.15)	146.5 ± 7.7 (0.93)	104.6 ± 8.5 (1.05)	104.6 ± 8.5 (1.05)
	1500	28.0 ± 2.8 (1.18)	34.0 ± 2.8 (1.51)*	134.5 ± 3.5 (0.95)	113.0 ± 1.0 (0.71)	106.3 ± 25.4 (1.07)	116.3 ± 8.0 (1.17)
	5000	24.0 ± 4.2 (1.01)	20.5 ± 2.1 (0.91)	167.0 ± 4.2 (1.18)	112.0 ± 4.5 (0.71)	61.5 ± 0.5 (0.62)	61.66 ± 0.5 (0.62)
**5b**	50	36.5 ± 7.7 (1.54)	24.6 ± 1.1 (1.09)	132.0 ± 11.3 (0.93)	124.3 ± 4.7 (0.79)	115.3 ± 20.2 (1.02)	102.3 ± 1.1 (0.9)
	150	29.6 ± 4.0 (1.25)	28.6 ± 4.7 (1.27)	129.0 ± 1.4 (0.91)	131.3 ± 1.1 (0.83)	104.6 ± 8.0 (0.92)	95.3 ± 9.8 (0.84)
	500	25.3 ± 6.6 (1.07)	40.0 ± 1.4 (1.77)**	153.5 ± 13.4 (1.08)	142.5 ± 9.1 (0.90)	125.0 ± 15.5 (1.1)	88.6 ± 3.7 (0.78)
	1500	28.5 ± 0.7 (1.20)**	32.6 ± 1.5 (1.44)**	157.6 ± 4.0 (1.11)	112.0 ± 14.1 (0.71)	99.3 ± 1.5 (0.87)	102.3 ± 8.0 (0.90)
	5000	23.5 ± 4.9 (0.99)	37.0 ± 1.4 (1.64)**	143.5 ± 12.0 (1.01)	134.5 ± 10.6 (0.85)	106.3 ± 2.8 (106.3)	83.0 ± 8.5 (0.73)
**5c**	50	25.5 ± 0.7 (1.00)	29.0 ± 1 (1.28)**	175.5 ± 12.0 (1.24)	133.0 ± 4.2 (0.84)	104.0 ± 3.4 (0.92)	95.3 ± 5.5 (0.96)
	150	26.6 ± 5.0 (1.12)	24.3 ± 6.1 (1.08)	158.5 ± 9.1 (1.12)	151.0 ± 7.0 (0.96)	97.0 ± 3.4 (0.85)	83.6 ± 4.5 (0.84)
	500	18.5 ± 2.1 (0.78)	19.5 ± 2.1 (0.86)	186.5 ± 6.3 (1.32)*	127.5 ± 6.3 (0.81)	101.0 ± 13.0 (0.89)	95.0 ± 3.60 (0.95)
	1500	27.0 ± 4.3 (1.14)	19.6 ± 1.5 (0.87)	147.0 ± 2.8 (1.04)	116.0 ± 1.4 (0.73)	97.3 ± 13.8 (0.86)	47.0 ± 6.9 (0.47)
	5000	25.5 ± 0.7 (1.06)	25 ± 5.6 (1.11)	164.5 ± 2.1 (1.16)	127.0 ± 2.8 (0.80)	85.3 ± 3.05 (0.75)	44.0 ± 0.5 (0.44)
	C-	23.6 ± 0.5	22.5 ± 0.7	141.0 ± 8.4	157.00 ± 1.0	113.00 ± 15.5	99.00 ± 3.4
	C+	343.00 ± 11,00[e]	1378.00 ± 8.00[b]	1338.00 ± 11.00[e]	1428.00 ± 6,00[c]	1.014 ± 9.4[e]	1284.00 ± 6.9[d]

[a]0: distilled water used as extract diluent; Positive Control (C +): [b]4-nitro-o-phenylenediamine (10 μg/plate); [c]Sodium Azide (2.5 μg/plate); [d]Mitomycin C (0.5 μg/plate); [e]2AA-aminoanthracene (2.5 μg/plate). Significant difference (ANOVA): *P ≤ 0.05; ** P ≤ 0.01.

Table S4. Hemolytic activity of the compounds

Compound	HC_{10} (µg/mL)	HC_{50} (µg/mL)
**5a**	173.5	> 1024
**5b**	36.6	54.8
**5c**	142.9	262.1

HC_{10} and HC_{50} are the concentrations of compounds at which 10% or 50% hemolysis was observed.

Table S5. Resistance profile of the isolates by susceptibility testing using the automated Vitek®2 system

**Isolates**	Samples	Isolation location	Type of MRSA	Gene Mec A	Gene Nuc A	SCCmec	Resistance
**1**	**26** – 08035507002	Urine	CA-MRSA	+	+	IV	CFO; OXA; PEN
**7**	**101** – 38274402	Peritoneal fluid	HA-MRSA	+	+	I	CFO; ERI; OXA; PEN

Cefoxitin – CFO; Erythromycin – ERI; Oxacillin – OXA; Penicillin G – PEN;

5a

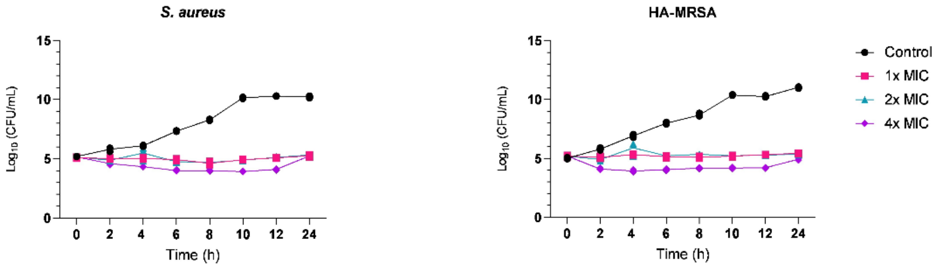

5b

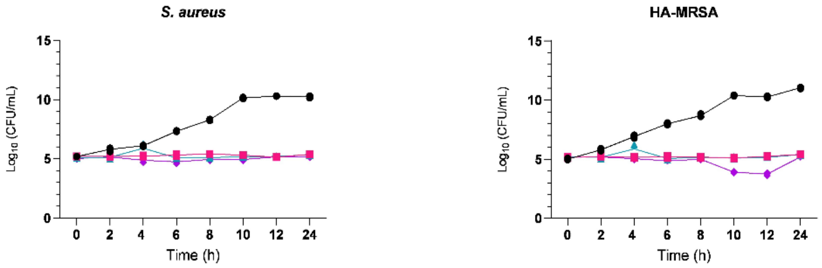

5c

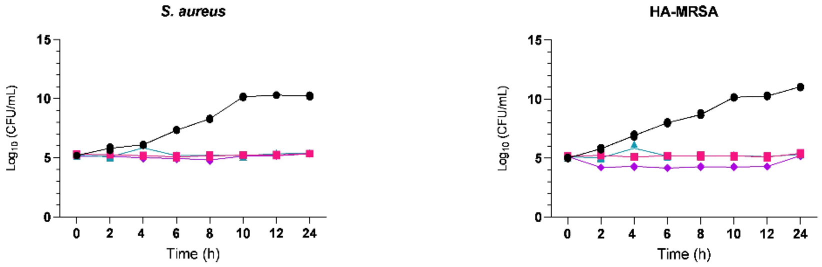

Figure S1. Effect of compounds on bacterial growth kinetics. The bacteria at a concentration of $1x10^8$ cells/mL and diluted 1:100 were initially inoculated in culture medium with concentrations equivalent to 4x, 2x and 1x MIC of compounds. Time points were recorded at 0, 2, 4, 6, 8, 10, 12 and 24 hours after inoculation. Data represent the mean ± standard deviation (N = 3). Omitted error bars indicate an SD value shorter than the size of the symbol presented in the plots.

5a

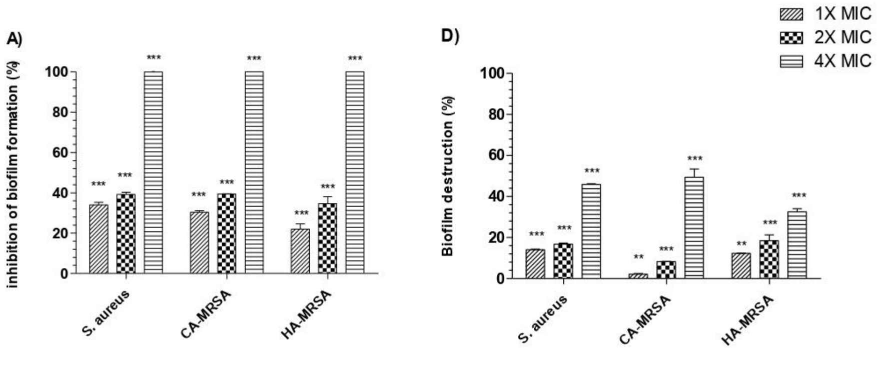

5b

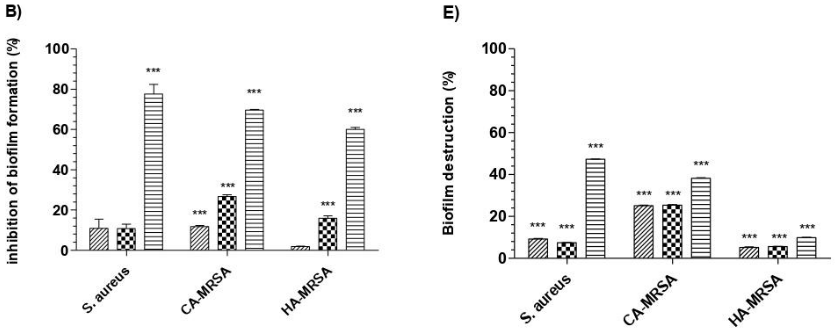

5c

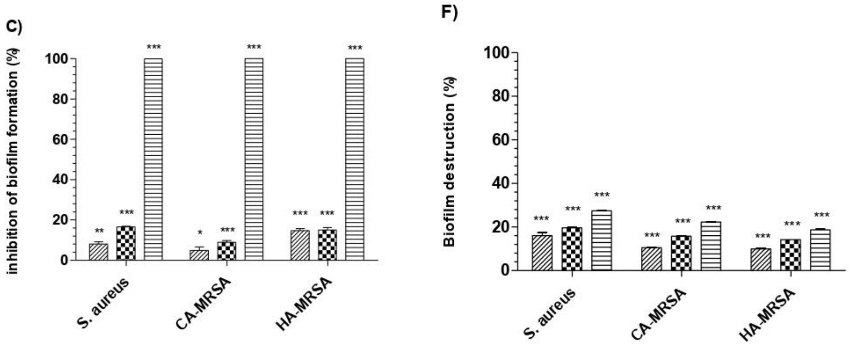

Figure S2. Potential of the compounds to inhibit (**A**, **B** and **C**) and to destroy (**D**, **E** and **F**) *S. aureus* (ATCC 29213), CA-MRSA and HA-MRSA biofilms evaluated according to biomass. *: $P \leq 0.05$; **: $P \leq 0.01$; ***: $P \leq 0.001$ according to ANOVA and Tukey's post-hoc. Each experiment included technical replicates. Omitted error bars indicate an SD value shorter than the size of the symbol presented in the plots.

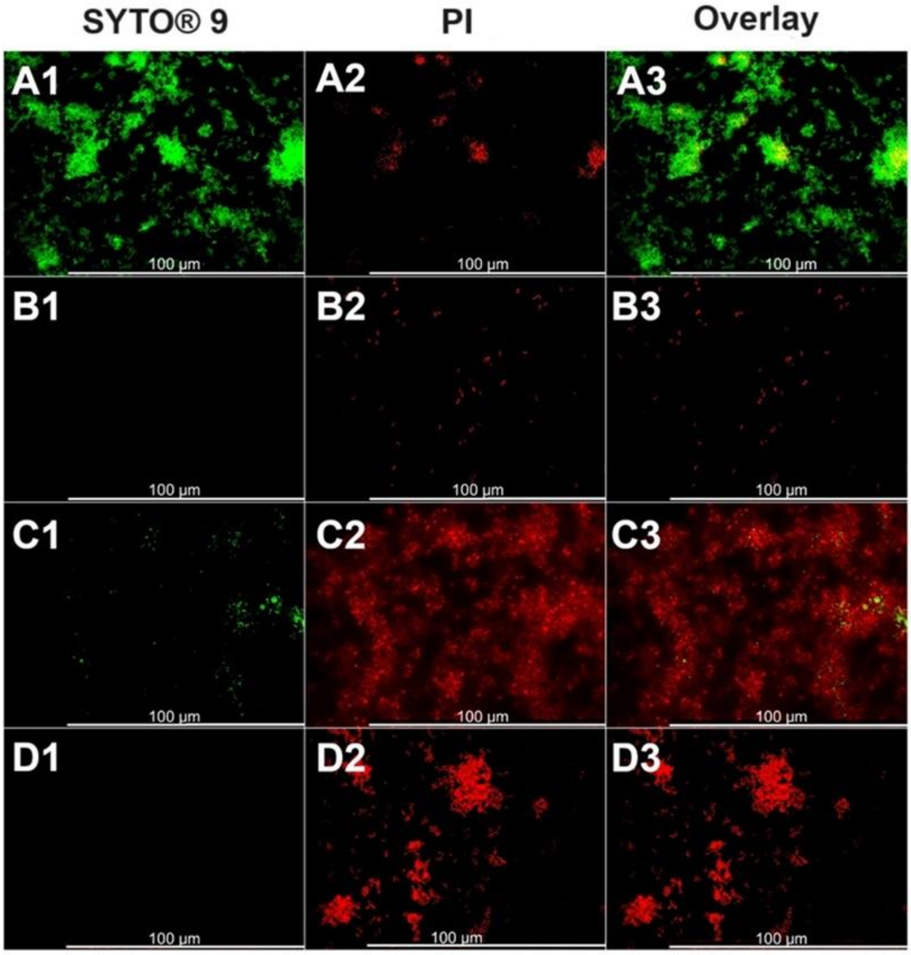

Figure S3. Cell viability of HA-MRSA biofilms by live/dead staining and fluorescence microscopy after treatments with compounds in the biofilm formation inhibition assay. The biofilms were cultivated in the presence of different concentrations of the compounds from an initial inoculum of 1 x 10^8 cells/mL for 48 hours. Living cells are stained fluorescent green and dead cells fluorescent red. **(A)** Control; **(B)** Treated at 1x MIC concentration of compound **5a**; **(C)** Treated at 1x MIC concentration of compound **5b**; **(D)** Treated at 1x MIC concentration of compound **5c**.

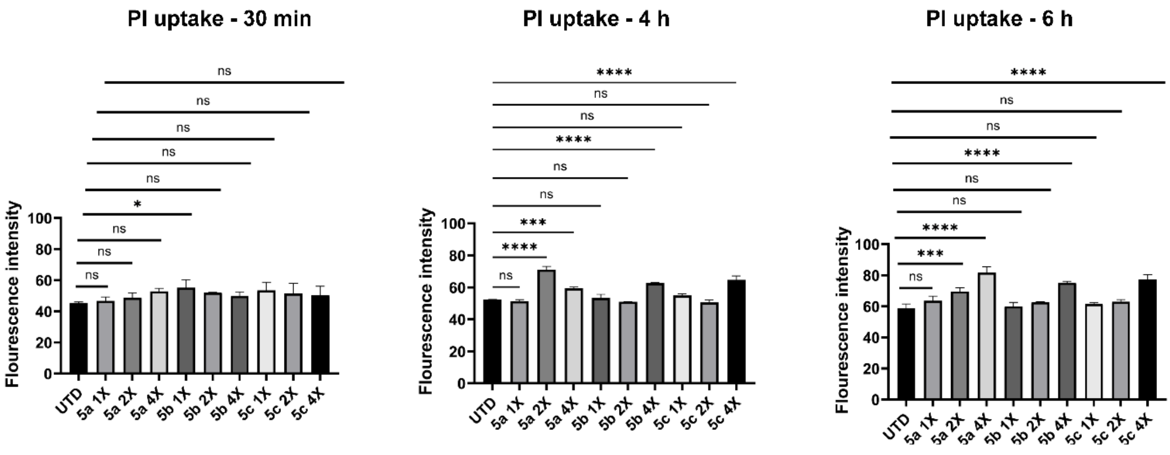

Figure S4. Inner membrane permeabilization evaluation by PI uptake against *S. aureus* JE2 displaying statistical analyses. Error bars report SEM. Each experiment included 3 biological replicates. P ≤ 0.0001; ns: not significant by One-Way ANOVA with Dunnett's multiple comparations test.

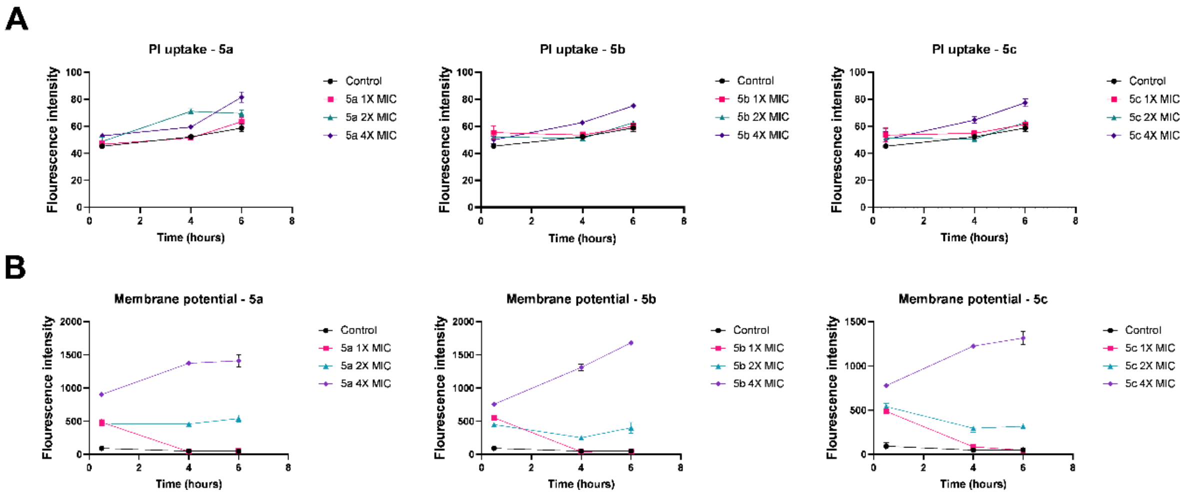

Figure S5. Kinect membrane activity investigation for the studied compounds at different concentrations against *S. aureus* JE2. **(A)** Inner membrane permeabilization evaluation by PI uptake; **(B)** Membrane potential analysis by the voltage sensitive fluorescent dye [DiSC3(5)].

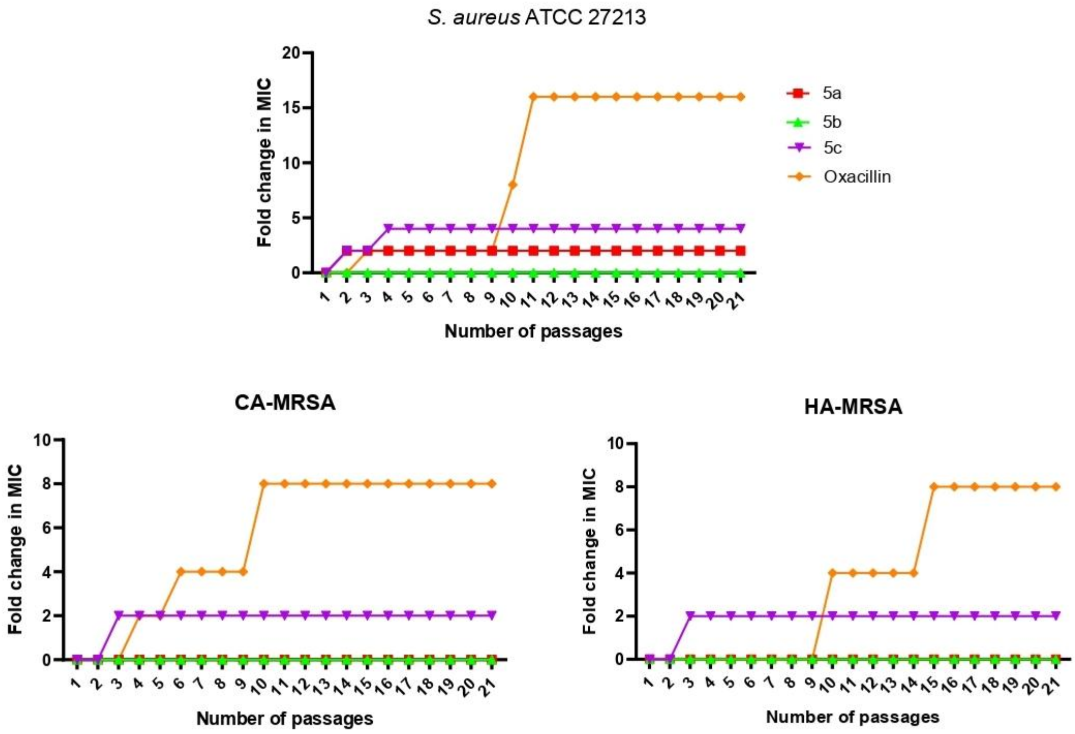

Figure S6. Study of resistance development in 21 days. The bacterial strains were cultivated in MHB at a concentration of ¼ MIC of the compounds. The broth microdilution assay was employed to determine the MIC value after each passage during the 21-day period.

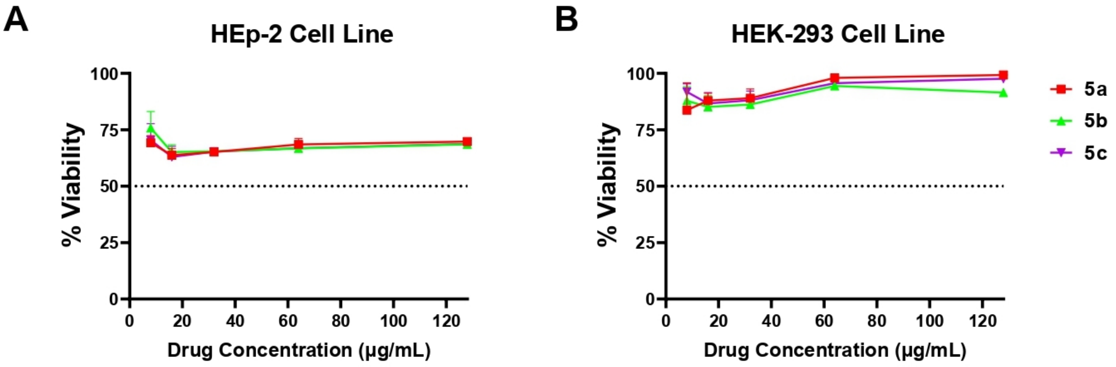

Figure S7. Viability of **5a**, **5b** and **5c** against HEp-2 **(A)**, HEK-293 **(B)** cell lines. Error bars report SEM (N = 3). Each experiment included technical replicates. Omitted error bars indicate an SD value shorter than the size of the symbol presented in the plots.
